# Broad protective RBD heterotrimer vaccines neutralize SARS-CoV-2 including Omicron sub-variants XBB/BQ.1.1/BF.7

**DOI:** 10.1371/journal.ppat.1011659

**Published:** 2023-09-18

**Authors:** Yanfang Zhang, Xinrui Kang, Sheng Liu, Pu Han, Wenwen Lei, Ke Xu, Zepeng Xu, Zhengrong Gao, Xuemei Zhou, Yaling An, Yuxuan Han, Kefang Liu, Xin Zhao, Lianpan Dai, Peiyi Wang, Guizhen Wu, Jianxun Qi, Kun Xu, George F. Gao

**Affiliations:** 1 CAS Key Laboratory of Pathogen Microbiology and Immunology, Institute of Microbiology, Chinese Academy of Sciences, Beijing, China; 2 Savaid Medical School, University of Chinese Academy of Sciences, Beijing, China; 3 Cryo-EM Center, Southern University of Science and Technology, Shenzhen, China; 4 NHC Key Laboratory of Biosafety, National Institute for Viral Disease Control and Prevention, Chinese Center for Disease Control and Prevention, Beijing, China; 5 Faculty of Health Sciences, University of Macau, Macau SAR, China; 6 Shenzhen Institute of Advanced Technology, Chinese Academy of Sciences, Shenzhen, China; 7 Shenzhen Children’s Hospital, Shenzhen, China; 8 School of Life Sciences, Hebei University, Baoding, China; 9 Research Network of Immunity and Health (RNIH), Beijing Institutes of Life Science, Chinese Academy of Sciences, Beijing, China; University of Colorado Denver, UNITED STATES

## Abstract

SARS-CoV-2 variants with severe immune evasion are a major challenge for COVID-19 prevention, especially the circulating Omicron XBB/BQ.1.1/BF.7 strains. Thus, the next-generation of broad-spectrum vaccines are urgently needed. Previously, we developed a COVID-19 protein subunit vaccine, ZF2001, based on the RBD-homodimer as the immunogen. To adapt SARS-CoV-2 variants, we developed chimeric RBD-heterodimers to induce broad immune responses. In this study, we further explored the concept of tandem RBD homotrimer and heterotrimer. Prototype SARS-CoV-2 RBD-homotrimer, prototype-Delta-BA.1 (PDO) RBD-heterotrimer and Delta-BA.2-BA.5 (DBA2BA5) RBD-heterotrimer were designed. Biochemical and cryo-EM structural characterization demonstrated total epitope exposure of the RBD-trimers. In mouse experiments, PDO and DBA2BA5 elicited broad SARS-CoV-2 neutralization. Potent protection against SARS-CoV-2 variants was observed in challenge assays and was correlated with neutralizing antibody titer. This study validated the design strategy of tandem RBD-heterotrimers as multivalent immunogens and presented a promising vaccine candidate, DBA2BA5, eliciting broad-spectrum immune responses, including against the circulating XBB/BF.7/BQ.1.1.

## Author summary

SARS-CoV-2 variants continue to emerge and circulate. Omicron sub-variants show severe evasion of the immune response induced by early-approved vaccines. Bivalent mRNA vaccines (prototype and Omicron BA.5) have been approved for use in the United States and other countries. However, the co-circulation of SARS-CoV-2 variants (such as BA.5.2, BF.7, BQ.1.1 and XBB) poses a challenge to disease control. The next-generation of COVID-19 vaccines need to induce potent and broad-spectrum immune responses. Here, we demonstrate a design strategy for trivalent SARS-CoV-2 vaccines with RBD-heterotrimers, which are single protein molecules without any linker sequences or trimerization domain. Biochemical and cryo-EM structural characterization demonstrated the total epitope exposure of RBD-trimers. Animal experiments showed that RBD-heterotrimers induce broad-spectrum protective immune responses. As a proof-of-concept, this trivalent immunogen design strategy is promising and practical for preventing the continuous circulation of SARS-CoV-2. This study provides guidance for future vaccine design against coronavirus and other viruses such as the influenza and dengue viruses.

## Introduction

SARS-CoV-2 continually evolves into new variants, bringing severe challenge to COVID-19 pandemic prevention [1,2]. The variants gained dominance one after another, particularly the Delta and Omicron variants of concern (VOCs) that circulate globally. The Omicron VOC carries multiple mutations on its receptor binding domain (RBD) of the spike (S) protein and displays extensive immune evasion from the developed therapeutic antibodies and vaccines [3–7]. More concerningly, emerging Omicron sub-variants display even further immune escape, greatly narrowing available countermeasures, especially the co-circulating Omicron BA.2 sub-variant XBB and the BA.5 sub-variants BF.7 and BQ.1.1 [7–9]. Omicron XBB nearly completely escapes the antibody activity induced by COVID-19 vaccines and neutralizing antibody drugs [10,11]. Thus, the development of broad-spectrum vaccines is urgently needed.

SARS-CoV-2 uses its S protein to recognize the host receptor, angiotensin-converting enzyme (ACE2), and mediate cell entry [12,13]. Most of the SARS-CoV-2 neutralizing antibodies are induced by the RBD of the S protein, rendering the RBD a significant target for the development of vaccines [14]. Previously, we designed immunogens of tandem repeat RBD-dimers as vaccines for betacoronaviruses [15], which were further developed for the COVID-19 protein subunit vaccine ZF2001 [16,17]. ZF2001 has received conditional marketing authorization or emergency use authorization in China, Uzbekistan, Indonesia, Columbia, Kenya and Belarus. Thereafter, due to the emergence and circulation of SARS-CoV-2 variants with immune evasion [5], we further designed heterologous chimeric RBD-dimers that induce broad immune responses against SARS-CoV-2. The concept of rapidly updating immunogens based on the chimeric RBD-dimer approach was validated [18]. Furthermore, the previous studies lead to important questions: how would the design strategy of tandem-repeat RBD and heterologous tandem RBD immunogens perform on RBD-trimers, and what are the immune responses and protection efficacies induced by the tandem RBD-trimers?

The SARS-CoV-2 trimeric RBD has been exploited as antigens in both mRNA and protein subunit vaccine candidates [19–24]. However, trimerization domains, such as the exogenous bacteriophage T4 fibritin foldon domain or the heptad repeats (HRs) of SARS-CoV-2 itself, are commonly used to produce self-assembled RBD-homotrimer protein. However, the true structure of the trimeric RBD has not yet been observed by either crystallography or cryo-electron microscopy (cryo-EM). In this study, we designed tandem RBD homotrimer and heterotrimers, determined the antigen integrity, resolved the structures of RBD-trimers by cryo-EM, analyzed their immunogenicity and demonstrated the protective efficacy in mice. This study validated the design concept of tandem RBD homotrimer and heterotrimer immunogens and demonstrated a promising COVID-19 vaccine candidate with broad spectrum activity, including against the circulating XBB, BF.7 and BQ.1.1 variants.

## Results

### Design and characterization of tandem RBD-trimer immunogens

**Fig 1:**
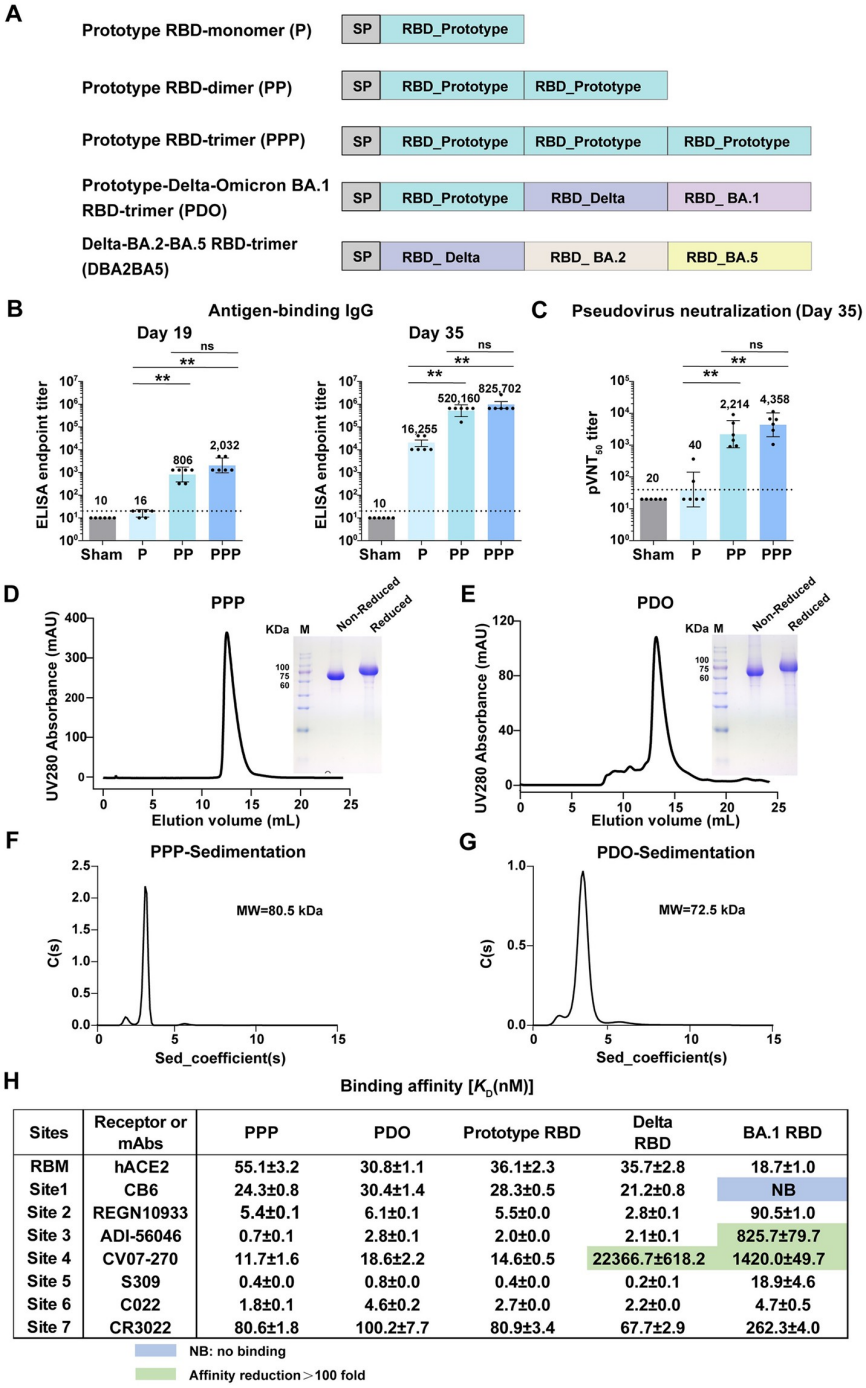
Design and characterization of tandem RBD homotrimer and heterotrimer. (A) Schematic diagram of the prototype SARS-CoV-2 RBD monomer (P), prototype RBD homodimer (PP), prototype RBD homotrimer (PPP), prototype-Delta-Omicron BA.1 tandem RBD heterotrimer (PDO) and Delta-BA.2-BA.5 tandem RBD heterotrimer (DBA2BA5). SP, signal peptide. (B-C) Groups of 6- to 8-weeks-old female BALB/c mice (n = 6) were immunized with two doses of 2 μg P, PP or PPP using SWE as adjuvant, 21 days apart. PBS plus adjuvant was given as the sham control. Sera were collected at 19 and 35 days after the first immunization. Endpoint titers of antigen-binding IgG in murine sera were measured by ELISA for each vaccine group by coating the antigen protein used for immunization. Neutralizing antibody titers were detected with VSV-based pseudotyped virus displaying the SARS-CoV-2 D614G spike protein. The values are the GMT ± 95% confidence interval (CI). The horizontal dashed line indicates the limit of detection (LOD). p values were analyzed with two-tailed Mann-Whitney tests (ns, p > 0.05; *p < 0.05; **p < 0.01). (D-E) Analytical gel filtration profiles of PPP (D) and PDO (E). The gel filtration was performed with a Superdex 200 Increase 10/300 GL column. The 280-nm absorbance curves are shown. SDS-PAGE analysis was conducted in non-reducing and reducing conditions. (F-G) Ultracentrifugation sedimentation profiles of PPP (F) and PDO (G). (H) Binding affinities of antigens bound to hACE2 and representative mAbs targeting seven major sites [7]. Light blue indicates no binding (NB). The ones with affinity reductions > 100-fold are colored in green.

To assess the concept of SARS-CoV-2 tandem RBD-trimer immunogens, we first designed a homotrimer composed solely of the prototype SARS-CoV-2 RBD (PPP) (Fig 1A). The PPP protein was expressed in Expi293F cells and purified. Then, gel filtration, gel electrophoresis and sedimentation velocity analytical ultracentrifugation assays were conducted, demonstrating that the purified PPP protein was stable as a single trimer-sized protein (molecular weight around 75 kDa) without cleavage (Fig 1D and 1F).

To evaluate the immunogenicity of the PPP antigen, BALB/c mice were immunized with two doses (21 days apart) of PPP protein vaccine adjuvanted with Sepivac SWE (hereafter referred to as SWE), a squalene-based oil-in-water adjuvant [25,26]. Prototype SARS-CoV-2 monomeric RBD (P) and dimeric RBD (PP) were used as comparisons. Blood samples were collected at 19 and 35 days post immunization for antibody titration. Endpoint titers of antigen-binding IgG from murine sera were measured by ELISA for each vaccine group by coating the antigen protein used for immunization. The results indicated that the PPP protein elicited significantly higher antigen-binding IgG and pseudovirus neutralizing antibody titers than the P protein. The levels of antibody responses induced by PPP protein were not significantly better than the PP protein (Fig 1B and 1C). These results demonstrated that the SARS-CoV-2 tandem RBD homotrimer protein was structurally stable and highly immunogenic.

Next, we tested the heterologous tandem connection strategy on the RBD-trimer and constructed PDO composed of the RBDs from prototype, Delta and Omicron (BA.1) (Fig 1A and S1 Fig). Each RBD contains eight cysteines and forms four disulfide bonds (S1 Fig). After expression and purification, the PDO protein was also demonstrated to be stable as a trimer-sized molecule (~75 kDa) (Fig 1E and 1G). Nonreduced proteins are smaller than reduced proteins by SDS-PAGE analysis because the reduced proteins are less compact and slowly migrate in the gel compared to the nonreduced proteins with intact disulfide bonds (Fig 1D and 1E).

To further study the epitopes displayed by the tandem RBD-trimer proteins, surface plasmon resonance (SPR) assays were performed. The binding affinities of PPP and PDO to human angiotensin-converting enzyme 2 (hACE2) and representative monoclonal antibodies targeting the seven major antigenic sites of the SARS-CoV-2 RBD [7,27] were analyzed. Monomeric SARS-CoV-2 RBD proteins from the prototype, Delta and Omicron BA.1 strains were also included as comparisons. We found that PPP and PDO bound to hACE2 with comparable affinities to those of the prototype RBD (Fig 1H and S2 Fig). CV07-270 (site RBD-4) was significantly evaded by both the Omicron BA.1 RBD and Delta RBD. In addition, the BA.1 RBD also lost binding ability to CB6 (site RBD-1) and showed largely reduced binding affinity to ADI-56046 (site RBD-3). As expected, the binding affinity profiles of PPP and PDO were similar to the prototype RBD, indicating that the major epitopes were fully displayed on PPP and PDO (Fig 1H and S2 Fig).

During preparation of this manuscript, new variants emerged, including Omicron sub-variants that display extraordinary immune escape. After the proof of concept with this heterotrimeric RBD design, we found that this vaccine does not provide good neutralization to continually emerging Omicron sub-variants. Thus, we immediately applied the strategy to develop a vaccine candidate as Delta-BA.2-BA.5 (DBA2BA5) for broader neutralization (Fig 1A). This more recent work is described in a later section.

### Cryo-EM structural analysis of RBD-trimers

**Fig 2:**
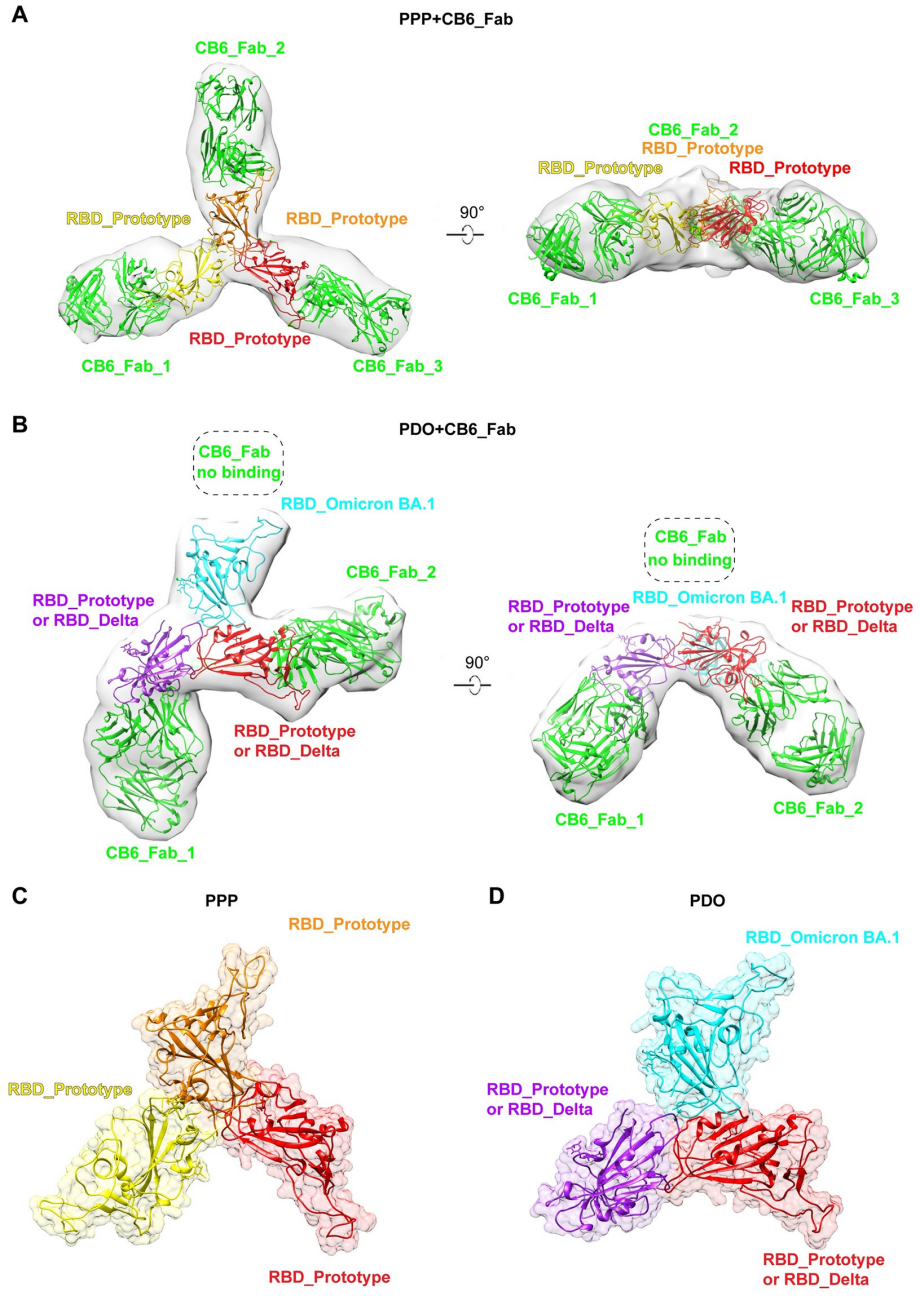
Structural characterization of RBD-trimers bound to CB6 Fabs. (A) Density map of PPP bound to three CB6 Fabs, into which the atomic models of the prototype RBD/CB6 complex (PDB:7C01) were fitted and rebuilt. (B) Density map of PDO bound to two CB6 Fabs, into which the atomic models of SARS-CoV-2 RBDs (Prototype RBD: 6LZG, Delta RBD: 7V8B, Omicron RBD: 7WBL) and the RBD/CB6 complex (PDB: 7C01) fitted and rebuilt. The Omicron BA.1 RBD does not bind to CB6. (C-D) Density map of PPP (C) and PDO (D) RBD-trimers individually fetched and presented.

For further analysis, we determined the cryo-EM structures of PPP or PDO in complex with a fragment antigen binding (Fab) of mAb CB6 [28] (S3 Fig). As the junctions of the RBDs have flexible N- and C-termini, the conformation of the PPP and PDO molecules was not uniform. After docking the previously reported molecular model of RBD/CB6 complexes, structures with low resolutions of 11.2 and 15 Å were determined for PPP/CB6 and PDO/CB6, respectively (S3 Fig). The PPP structure displays a rotationally symmetric conformation, with all three RBD/CB6 units in the same plane (Fig 2A). The RBDs stack together via their core motifs, exposing the external motifs (Fig 2C). Consistent with the fact that CB6 no longer binds to the Omicron BA.1 RBD [7], only two CB6 Fabs were observed to bind to PDO in the PDO/CB6 Fab complex, leaving one RBD unbound and more space for the RBDs to occupy (Fig 2B and S3 Fig). In addition, the prototype RBD and Delta RBD could not be differentiated in the PDO structure. Compared to the PPP/CB6 complex, the PDO/CB6 complex displays a more twisted conformation, with CB6 Fabs bending to the same direction, which might result from the unbalanced interactions of the CB6 Fabs (Fig 2B). The PDO complex also displays a roughly rotationally symmetric conformation, but is not as regularly shaped as the PPP (Fig 2D). It is also noteworthy that the structures only display one possible conformation due to the flexible sequences in the connections between RBDs. Nonetheless, these cryo-EM structures demonstrated that the trimeric RBD immunogens are well exposed.

### Immunogenicity of tandem RBD-trimer vaccines in mice

**Fig 3:**
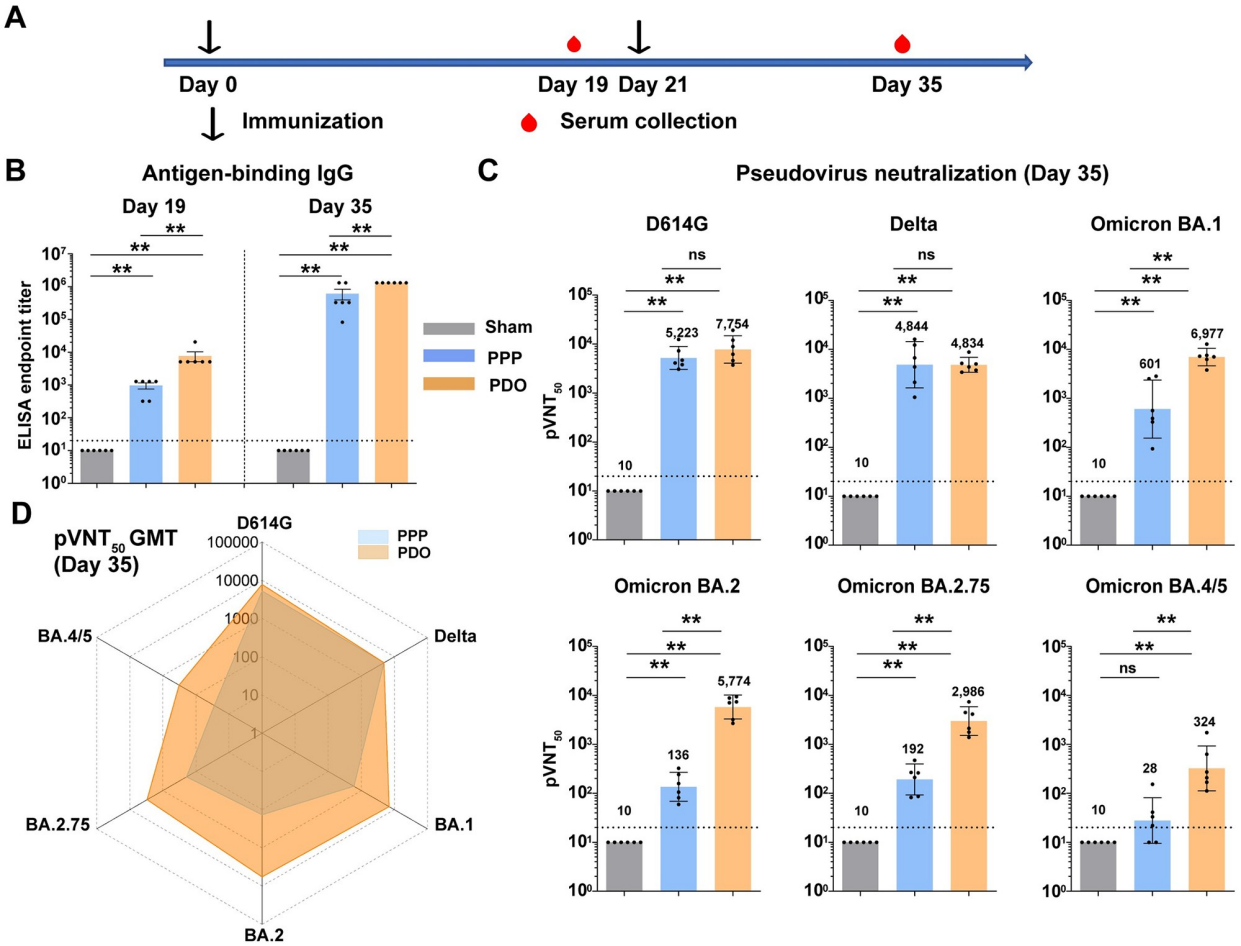
Immunogenicity of PPP and PDO in mice. (A) Time course of mouse experiments. Groups of 6- to 8-weeks-old female BALB/c mice (n = 6) were vaccinated with two doses of immunogen (2 μg) adjuvanted with SWE. PBS plus adjuvant was given as the sham control. Blood samples were collected at days 19 and 35. (B) Endpoint titers of antigen-binding IgG in murine sera were measured by ELISA for each vaccine group by coating the antigen protein used for immunization. A mixture of PPP and PDO proteins was coated for the sham group. (C) A panel of pseudotyped viruses was used to detect the pVNT_50_ for the sera collected at day 35. (D) Radar plot demonstrating the neutralization profile of sera elicited by two doses of PPP and PDO vaccine candidates against six SARS-CoV-2 pseudotyped viruses. For (B and C), shown are the GMT ± 95% CI. The horizontal dashed line indicates the LOD. p values were analyzed with two-tailed Mann-Whitney tests (ns, p > 0.05; **p < 0.01).

To evaluate the immunogenicity, groups of BALB/c mice at 6- to 8-weeks old were immunized with two doses (21 days apart) of 2 μg PPP or PDO protein vaccines adjuvanted with SWE. PBS mixed with SWE was applied as a sham control. Serum samples were collected at days 19 and 35 (Fig 3A). Antigen-binding IgG antibodies were analyzed and showed potent humoral immunity induced by both PPP and PDO (Fig 3B). PDO induced higher binding IgG titers than PPP after the first and second doses (Fig 3B). Next, the neutralizing spectra of mouse sera after two doses of RBD-trimer vaccines were further evaluated with pseudotyped viruses displaying SARS-CoV-2 D614G, Delta, and Omicron sub-variants BA.1, BA.2, BA.2.75, BA.4 and BA.5 (Fig 3C and 3D). BA.4 and BA.5 share identical S protein sequences and are thus designated BA.4/5. The GMTs of 50% pseudovirus neutralization titers (pVNT_50_) were analyzed. We found that both PPP and PDO elicited robust neutralizing activities against D614G and Delta variants with high pVNT_50_ titers, which were between 4834 and 7754 (Fig 3C and 3D). However, PPP-elicited murine sera showed rather low pVNT_50_ (GMTs from 136–601) against Omicron BA.1, BA.2 and BA.2.75 and a pVNT_50_ of 28 against Omicron BA.4/5. For mice that received the PDO vaccine, the pVNT_50_ GMTs of sera against BA.1, BA.2, BA.2.75 and BA.4/5 were 6977, 5774, 2986 and 324, respectively, which were much higher than PPP (Fig 3C and 3D). The radar plot clearly shows that PDO outperformed PPP in neutralizing against all of the tested variants, especially for the Omicron sub-variants, indicating a broad-spectrum neutralizing profile (Fig 3D).

### Protection efficacy of the PDO vaccine in mice

**Fig 4:**
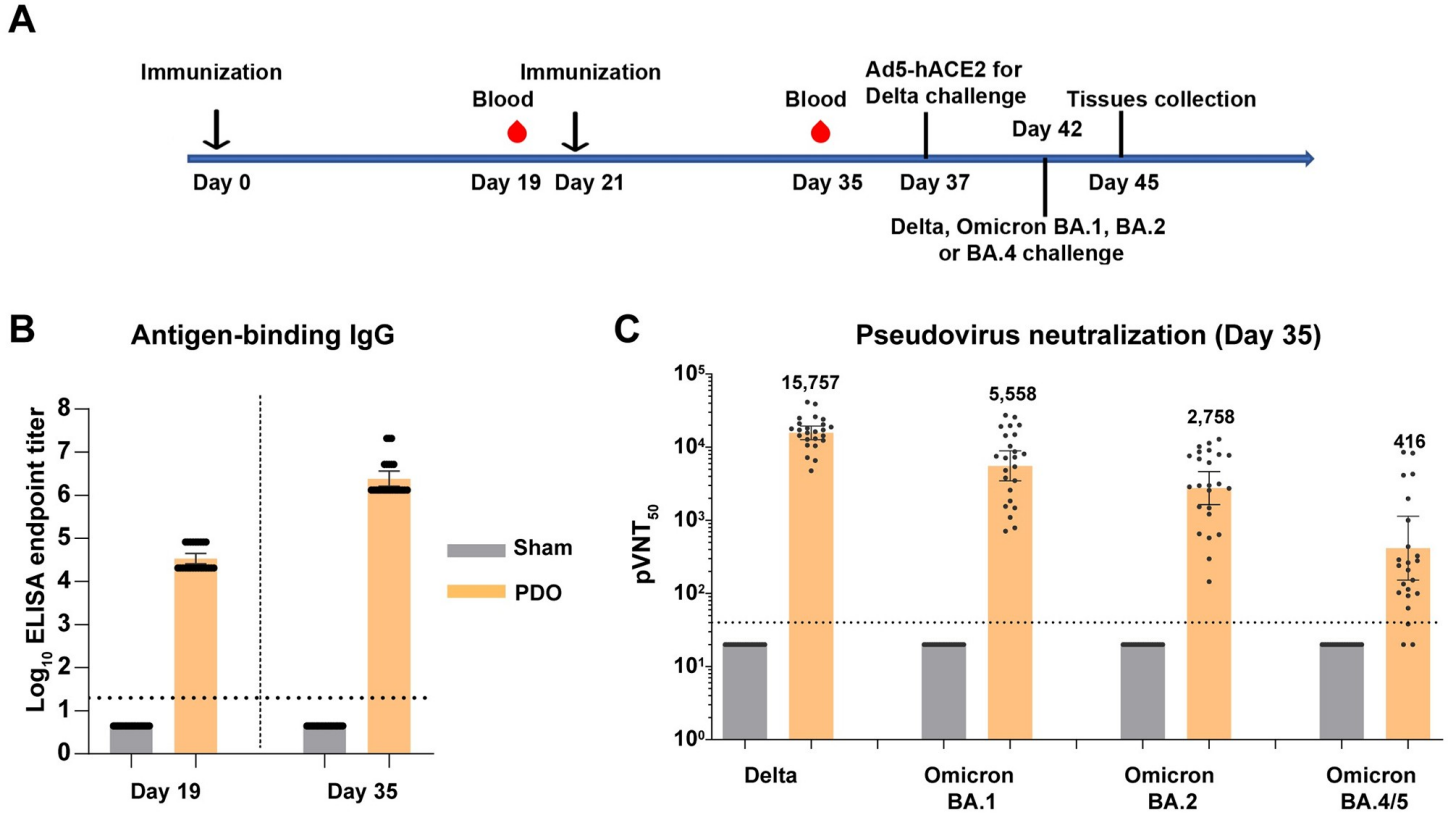
SARS-CoV-2 challenge studies. (A) Immunization and challenge schedule. Female BALB/c mice at 6- to 8-weeks-old were vaccinated with two doses of PDO adjuvanted of SWE. PBS plus adjuvant was given as the sham control. Sera were collected at days 19 and 35, respectively. For Delta VOC challenge, mice were transduced with Ad5-hACE2 via the intranasal route and infected with Delta VOC (NPRC 2. 192100004) five days later. For Omicron variants challenge, mice were directly infected with BA.1 (NPRC 2. 192100009), BA.2 (NPRC 2. 192100010) or BA.4 (NPRC 2. 192100012) via the intranasal route. Tissue was collected at three days post challenge. (B) Endpoint titers of PDO-binding IgG in murine sera were detected by ELISA. (C) Neutralizing antibody titers were detected with a panel of pseudotyped viruses displaying SARS-CoV-2 Delta, Omicron BA.1, Omicron BA.2 and Omicron BA.4/5 spikes. For (B and C), shown are the GMT ± 95% CI. The horizontal dashed line indicates the LOD.

To further explore the protective efficacy of the RBD-trimer vaccine, BALB/c mice receiving two doses of PDO were challenged by authentic SARS-CoV-2 infection (Fig 4A). The PDO-elicited murine sera were collected before the challenge for antibody titrations. Similar to the results from the above studies, high titers of antigen-binding IgG and neutralizing antibodies were observed (Fig 4B and 4C).

**Fig 5:**
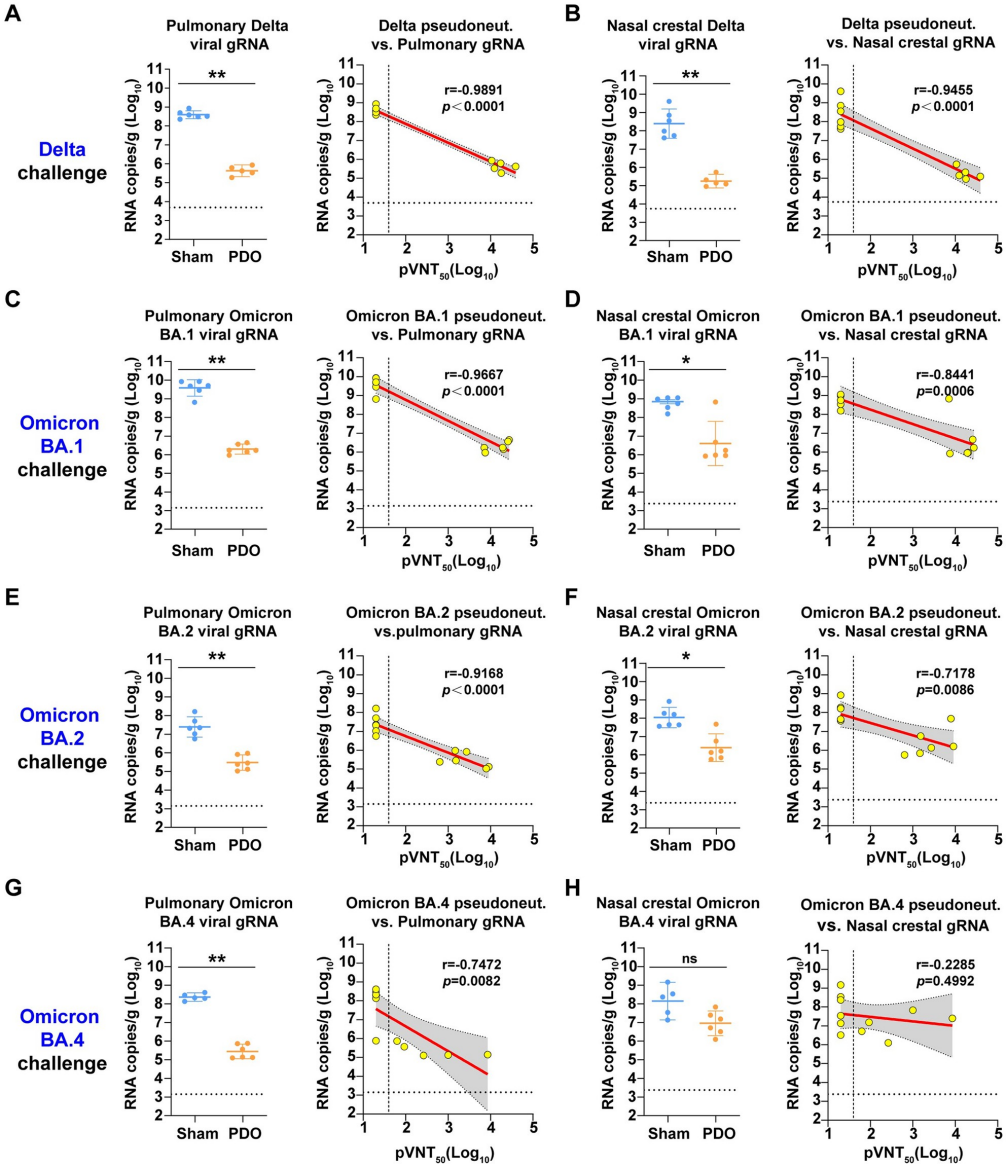
Protection efficacies of PDO against SARS-CoV-2 variants. (A, C, E, G) Pulmonary viral loads. Pulmonary viral gRNA levels of Delta (A), Omicron BA.1 (C), Omicron BA.2 (E) and Omicron BA.4 (G) were detected by qRT-PCR (left). Plots show correlations and corresponding two-sided p values between pVNT_50_ and pulmonary viral gRNA (right). (B, D, F, H) Nasal crestal viral loads. Nasal crestal viral gRNA levels of Delta (B), Omicron BA.1 (D), Omicron BA.2 (F) and Omicron BA.4 (H) were detected by qRT-PCR (left). Plots show correlations and corresponding two-sided p values between pVNT_50_ and nasal crestal viral gRNA (right). The values of viral gRNA levels are the GMT ± 95% CI. The horizontal dashed line indicates the LOD. p values were analyzed with two-tailed Mann-Whitney tests (ns, p > 0.05; *p < 0.05; **p < 0.01). For correlation analysis, red and gray lines indicate the linear regression line and 95% CI, respectively. r and p values represent Spearman’s correlation coefficients and corresponding two-sided p values, respectively.

BALB/c mice are not sensitive to Delta variant infection due to the low binding affinity between mouse ACE2 and Delta S protein, but they are sensitive to Omicron variants due to the increased mouse ACE2 affinity induced by the N501Y mutation of the S protein [29]. Therefore, mice challenged with the Delta variant strain were transduced with recombinant adenovirus expressing hACE2 (Ad5-hACE2) via the intranasal route five days before the Delta variant challenge. For mice challenged with Omicron BA.1, BA.2 and BA.4, no hACE2 was transduced. All mice were euthanized and necropsied at three days post-infection to quantify viral RNA in the upper (turbinate bones) and lower (lung) respiratory tract (Fig 4A). We found that, compared to sham groups, the average viral loads in lung samples were largely reduced in the PDO groups for all of the tested challenge SARS-CoV-2 strains (Fig 5A, 5C, 5E and 5G). The average viral loads in turbinate bones were also reduced by 1383-, 146-, 44- and 16-fold for PDO-vaccinated mice challenged with Delta, Omicron BA.1, BA.2 and BA.4, respectively, compared to sham-vaccinated mice (Fig 5B, 5D, 5F and 5H).

Correlation analyses between the pVNT_50_ and viral RNA load were conducted and demonstrated significantly negative correlations in lung tissues from mice challenged with Delta, BA.1, BA.2 and BA.4 (Fig 5A, 5C, 5E and 5G). In addition, in the nasal turbinates, Delta-, BA.1- and BA.2-challenged mice displayed significantly negative correlations (P<0.01), while no significant correlation was observed in BA.4- challenged mice. These results suggested that the PDO vaccine protected mice against SARS-CoV-2 Delta, Omicron BA.1, and BA.2 but not BA.4 infection in nasal turbinates (Fig 5B, 5D, 5F and 5H).

### Induction of broad-spectrum immune responses against variants including XBB and BQ.1.1 by DBA2BA5

**Fig 6:**
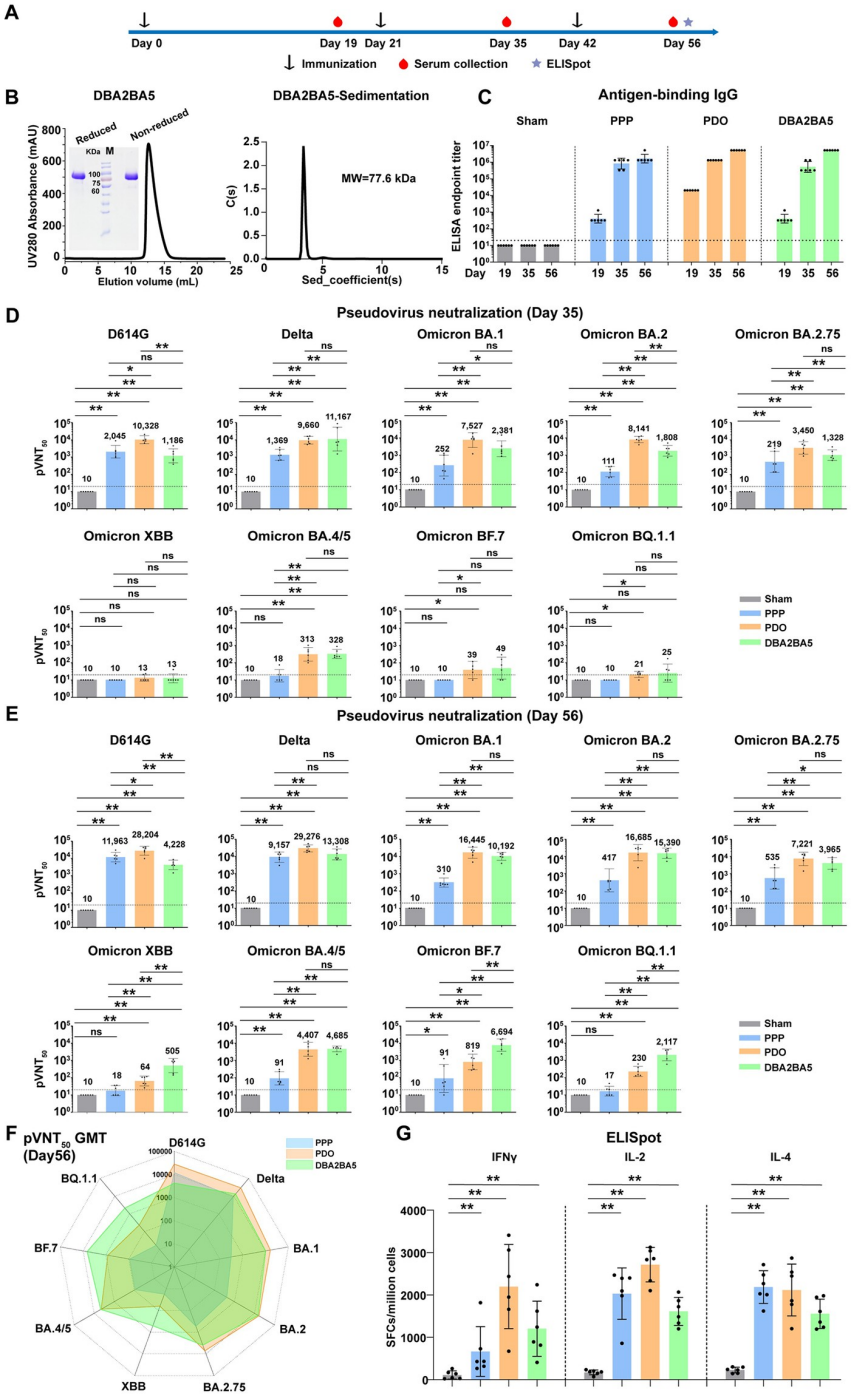
Design and evaluation of the DBA2BA5 RBD heterotrimer immunogen. (A) Time course of mouse experiments. Groups of 6- to 8-weeks-old female BALB/c mice (n = 6) were vaccinated with three doses of immunogen (2 μg) adjuvanted with SWE. PBS plus adjuvant was given as the sham control. Blood samples were collected at days 19, 35 and 56. (B) Analytical gel filtration and ultracentrifugation sedimentation profile of the DBA2BA5 tandem RBD heterotrimer. The gel filtration was performed on a Superdex 200 Increase 10/300 GL column. The 280-nm absorbance curves are shown. SDS-PAGE analysis was conducted in non-reducing and reducing conditions. (C) Endpoint titers of antigen-binding IgG in murine sera were measured by ELISA for each vaccine group by coating the antigen protein used for immunization. A mixture of PPP, PDO and DBA2BA5 proteins was coated for the sham group. (D-E) A panel of pseudotyped viruses was used to detect the pVNT_50_ for the sera collected at days 35 (D) and 56 (E). (F) Radar plot demonstrating the neutralization profile of sera elicited by three doses of PPP, PDO and DBA2BA5 vaccine candidates against nine SARS-CoV-2 pseudotyped viruses. (G) Evaluation of cellular immune responses. Mouse IFNγ, IL-2 and IL-4 ELISpot assays were performed. The values are means ± SEM. p values were analyzed with two-tailed Mann-Whitney tests (**p < 0.01). For (C, D and E), shown are the GMT ± 95% CI. The horizontal dashed line indicates the LOD. p values were analyzed with two-tailed Mann-Whitney tests (ns, p > 0.05; *p < 0.05; **p < 0.01).

Similar to PPP and PDO, the DBA2BA5 protein was expressed in Expi293F cells, purified and analyzed by gel filtration and gel electrophoresis assays (Fig 6B). To study the stability of DBA2BA5 protein, we stored the purified DBA2BA5 protein at 4°C for 0, 7, 14, 21 and 28 days, respectively. Then, the collected DBA2BA5 protein samples were analyzed by analytical ultracentrifugation assays. We observed that the DBA2BA5 protein is stable for at least four weeks at 4°C without aggregation or degradation (S4 Fig). These results demonstrated the high purity, correct molecular weight, and stability of the DBA2BA5 protein (Fig 6B and S4 Fig).

Next, the immunogenicity of DBA2BA5 was evaluated. Groups of female BALB/c mice were immunized with SWE-adjuvanted PPP, PDO or DBA2BA5. Three doses were given at 21-day intervals. Sera were collected after each administration to analyze the humoral immune responses. Spleens were collected at day 56 to analyze the cellular immune responses. The schedule is depicted in Fig 6A.

We measured the antigen-binding IgG titers by ELISA for each vaccine group by coating the antigen protein used for immunization. We found that antigen-binding IgG antibodies were robustly induced, and antibody titers increased following each vaccination (Fig 6C). In addition, the sera binding antibody activities to prototype, Delta, Omicron BA.1, BA.2, or BA.5 RBD monomer protein were tested and displayed cross-binding activities (S5 Fig). The serological neutralizing antibody activities induced by two and three doses of vaccination were evaluated. After the second vaccination, the DBA2BA5 vaccine induced high levels of pVNT_50_ against SARS-CoV-2 D614G (GMT, 1186), Delta (GMT, 11167), BA.1 (GMT, 2381), BA.2 (GMT, 1808) and BA.2.75 (GMT, 1328) (Fig 6D). The response to the boost with DBA2BA5 was not as robust as the response to PDO after the first boost. The pVNT_50_ against BA.4/5 induced by DBA2BA5 decreased to 328, which was similar to the PDO group. However, for XBB, BF.7 and BQ.1.1, two doses of PPP-elicited sera lost neutralizing activities. Two doses of PDO or DBA2BA5 induced low levels of pVNT_50_ against BF.7 and BQ.1.1, which were near the limit of detection (LOD) and did not induce neutralizing responses against XBB (Fig 6D).

The GMTs of pVNT_50_ were further increased after the third dose in all RBD-trimer groups (Fig 6E and S6 Fig). The fold change of the elicited antibodies against the early D614G *vs*. the other variants was analyzed in S7 Fig. In the PPP group, the neutralizing GMTs were ~10^4^ against the D614G and Delta variants, between 310 and 535 against BA.1, BA.2 and BA.2.75, approximately 10^2^ against BA.4/5 and BF.7, and decreased to below the LOD against BQ.1.1 and XBB (Fig 6E). The PDO-elicited serological neutralization profile was much broader than PPP. However, the neutralizing activities against BQ.1.1 and XBB were moderately elevated by the third dose but still at low levels (GMTs, XBB: 64; BQ.1.1: 230) (Fig 6E and 6F). After three-doses of DBA2BA5 vaccination, a balanced neutralizing activity profile was achieved, including against the circulating XBB, BF.7 and BQ.1.1 variants. The pVNT_50_ were between 3965 and 15390 against D614G, Delta, BA.1, BA.2, BA.2.75 and BA.4/5. In addition, the sera effectively neutralized XBB (GMT, 505), BF.7 (GMT, 6694), and BQ.1.1 (GMT, 2117) (Fig 6E and 6F).

To study the durability of the antibody responses induced by the DBA2BA5 vaccine, a group of female BALB/c mice (n = 6) at 6- to 8-weeks old were immunized with three doses (21 days apart) of 2 μg DBA2BA5 protein vaccine adjuvanted with SWE. PBS mixed with SWE was applied as a sham control. Serum samples were collected at 21 and 120 days after the last vaccination. The neutralizing activities were measured and revealed that the titers slightly decreased by 1.2- to 2.5-fold. These results indicate that the humoral immune responses were largely preserved 4 months post-vaccination (S8 Fig).

In addition to humoral immune responses, vaccine-elicited cellular immunity plays a crucial role in preventing COVID-19 [30]. Therefore, enzyme-linked immunospot (ELISpot) assays were performed to evaluate the cellular immune responses. Murine splenocytes were stimulated with SARS-CoV-2 peptide pools covering prototype, Delta, and Omicron BA.1 RBDs. High production of IFNγ, IL-2, and IL-4 cytokines were observed in all of the RBD-trimer groups, suggesting the induction of potent and multifunctional cellular immunity (Fig 6G). In addition, we observed that the PDO group showed the highest levels of cellular responses. The cellular responses induced by DBA2BA5 were lower than PDO, likely due to immune evasion by BA.2 and BA.5 (Fig 6G).

The above studies demonstrated that these RBD-trimers induced both humoral and cellular immune responses. Particularly, DBA2BA5 elicited broad and balanced immune responses against SARS-CoV-2 variants, including the co-circulating SARS-CoV-2 variants XBB, BF.7 and BQ.1.1.

## Discussion

In this study, we designed a prototype SARS-CoV-2 tandem-repeat RBD-homotrimer, tandem prototype-Delta-Omicron BA.1 (PDO) RBD-heterotrimer, and Delta-BA.2-BA.5 (DBA2BA5) RBD-heterotrimer. No exogenous sequence was used as a linker between the RBDs. The antigen integrity, stability and cryo-EM structures of the RBD-trimers were determined. The immunogenicity of these RBD-trimers was evaluated in mice and revealed that DBA2BA5 elicited broad-spectrum neutralizing activities against SARS-CoV-2 variants, including the globally circulating XBB, BQ.1.1 and BF.7 strains. Consistently, authentic virus challenge studies demonstrated the potent protection efficacy of RBD-heterotrimer from infection of multiple SARS-CoV-2 variants, which was correlated with neutralizing antibody titers. This study further validated the concept of tandem RBD-trimers and offers guidance for future vaccine design.

The SARS-CoV-2 RBD monomer vaccine cannot elicit broad-spectrum antibody immune responses against multiple strains. Antibody responses elicited by early strains, such as D614G, Alpha, Beta, and Delta, can be evaded by Omicron sub-variants. By contrast, Omicron sub-variant-elicited antibodies also display poor neutralizing activities against early strains. Therefore, a mixture of the variant monomers can induce relatively broad immunity. Previously, we demonstrated that RBD homodimers induce higher levels of humoral immunity than RBD monomers [15], and RBD heterodimers induce broad immunity against variants [18]. This study shows that RBD homotrimers are robustly immunogenic and induce similar levels of antibody responses compared to RBD homodimers. The RBD heterotrimer is a trivalent immunogen that can be updated with the sequences of co-circulating strains. We analyzed the cryo-EM structure of RBD trimers and found that the RBDs stack together, exposing the external RBM motifs, which potentially improves immune-focusing on the immunodominant epitopes (Fig 2). In addition, compared to a mixture of the variant monomers, the RBD-trimer may elicit cross-linking of B cell receptors on B cells for stronger stimulation. Cellular immunity plays a crucial role in preventing the disease caused by SARS-CoV-2. The mutations in variants could affect the recognition of T-cell epitopes [30,31]. RBD heterotrimers contain more T-cell epitopes than RBD homotrimers, RBD dimers, and monomers, which is advantageous for eliciting broad T-cell immune responses.

Omicron sub-variants are co-circulating worldwide and show severe escape to immune responses induced by COVID-19 vaccines based on the prototype SARS-CoV-2 sequence, especially XBB and BQ.1.1 [10,11]. Phylogenetic analyses show that XBB is closely related to BA.2, and BF.7/BQ.1.1 are closely related to BA.5 [10]. The DBA.2BA.5 RBD-trimer immunogen was designed before the emergence of XBB and BQ.1.1, suggesting that the tandem RBD-heterotrimer strategy is promising and practical for preventing infection by future variants.

We observed that three-dose immunization is necessary to induce strong neutralizing activities against some variants. This phenomenon can be explained by the immunodominant epitopes of immunogens. Two doses of vaccine can elicit strong immune responses by activating the immunodominant epitopes of the immunogen but not non-immunodominant epitopes. Moreover, after a third dose of immunization, the pre-induced antibodies in the body will bind to the immunodominant epitopes of the antigen protein. Therefore, the rare epitopes would be stimulated to generate potent immune responses.

The coronavirus RBD is a major target for the development of therapeutic antibodies and vaccines due to the induction of high-quality and functionally relevant antibodies and T-cell responses [14,32,33]. The constant emergence and circulation of immune-evasive SARS-CoV-2 variants and the potential threat of zoonotic coronavirus spillover demonstrates the need for a broad-spectrum vaccine. The tandem RBD-heterotrimer immunogen will allow broader coverage of SARS-CoV-2 variants and inclusion of other coronaviruses, such as SARS-CoV and zoonotic coronaviruses. It will also be a promising strategy for pan-sarbecovirus vaccines, which we will further explore.

The SWE-adjuvanted RBD trimer vaccines elicit balanced cellular immune responses, including helper T (Th)1 cytokine (IFNγ and IL-2)- and Th2 cytokine (IL-4)-secreting cells. Previously, the protein subunit vaccine ZF2001, containing the RBD-dimer protein and aluminum hydroxide adjuvant, induced both Th1 (IFNγ and IL-2) and Th2 (IL-4 and IL-5) cell responses in clinical trials. Safety studies show that ZF2001 is safe and well-tolerated [16,17]. Therefore, the cellular immune responses induced by SWE-adjuvanted RBD trimers are speculated to be safe, though this needs to be verified in preclinical studies. In addition, we have not studied the correlation between cellular immunity and protectivity in challenge experiments. The published literature reveals that T-cell responses alone can provide partial protection. Lee *et al*. report that a vaccine containing 20 synthesized peptides targeting SARS-CoV-2 S and nucleocapsid protein with an RNA adjuvant elicits multifunctional T-cell responses and alleviates pulmonary pathology in hACE2 transgenic mice after challenge with SARS-CoV-2 [34]. Zhuang *et al*. also report that T-cell vaccination alone partially protects mice against severe disease caused by SARS-CoV-2 infection [35].

To study the functional properties of antibodies induced by the vaccine candidates, single B cells can be isolated from vaccinated mice to test monoclonal antibody binding and neutralizing activities. Previously, we studied the functional property of Zika virus vaccine candidate-induced antibodies in a mouse model [36]. The antibody repertoire elicited by the RBD trimers will likewise be studied in further research.

The tandem RBD-trimer design strategy allows RBDs from three different strains to be incorporated into one construct, alleviating the formulation of independent constructs of monomeric RBDs. However, most dominant SARS-CoV-2 Th cell and cytotoxic T lymphocyte (CTL) epitopes are located outside the RBD. Targeting these Th/CTL epitopes can play a pivotal role in protecting against moderate-severe disease and death and likely in the prevention of long-haul COVID [37–39]. The published literature demonstrates the protective immunity induced by T-cell epitopes. A multi-epitope vaccine (UB-612) containing the S1-RBD-sFc protein and promiscuous peptides representing sarbecovirus conserved Th/CTL epitopes on the nucleocapsid (N), membrane (M), and S2 proteins synergistically elicits broadly recognizing and durable B-cell and Th1-oriented T-cell immune responses in participants [39,40]. In addition, a universal influenza vaccine candidate based on nanoparticles presenting highly conserved epitopes induces long-lasting and broad humoral and cellular immunity against influenza in the murine model [41]. Another influenza vaccine candidate, FLU-v, containing four short peptides targeting influenza conserved internal proteins M1, NPA, NPB, and M2 and adjuvanted by Montanide ISA-51 induces cross-reactive cell-mediated immunity and provides protection against mild-to-moderate influenza disease in human participants [42,43]. Therefore, a combination of the RBD heterotrimer and SARS-CoV-2 conserved Th/CTL epitopes would likely result in synergistic humoral B cell and cellular T cell immunity, allowing broad coverage of SARS-CoV-2 variant strains. This is a potential and promising approach and deserves further exploration.

One limitation of this study is that the viral loads were not measured by plaque assays (Fig 5). Plaque assay data represent the viable viral loads, which is better to reveal the protection efficacy. Our data show that compared to the sham groups, the viral RNA levels of nasal turbinates from PDO-vaccinated mice were reduced by 1383-, 146-, 44-, and 16-fold for Delta, Omicron BA.1, BA.2, and BA.4 challenge, respectively. These results are consistent with other studies that observed that BA.4 displays the highest transmissibility among these four strains, followed by BA.2, BA.1, and Delta, respectively [44–47]. In addition, qRT-PCR is a widely used method to determine the viral titers in developing SARS-CoV-2 vaccines and drugs.

A large number of humans have already received COVID-19 vaccines. As SARS-CoV-2 shows significant diversity, the next generation of COVID-19 vaccines should induce broad and balanced immunity against diversified strains. In October 2022, both Pfizer-BioNTech and Moderna received emergency use authorizations by the US FDA for COVID-19 bivalent mRNA vaccines with equally dosed ancestral and BA.4/5 mRNAs. However, a preprint article reports that the bivalent vaccine demonstrates no advantage over the original formula [48]. One possible reason is that the efficiency of S protein production by BA.4/5 mRNA is lower than the prototype mRNA. Precisely controlling antigen protein composition is an advantage for protein subunit vaccines.

Original antigenic sin is a phenomenon in which the previous immune background can limit the antibody responses induced by subsequent related antigens [49]. Ju *et al*. evaluate the antibody responses in prototype strain-based inactivated vaccine-immunized cohorts. They find that although BA.2 breakthrough infections induce a certain cross-neutralization activity against later Omicron subvariants, the improvement of variant-specific antibody responses is possibly limited by the original antigenic sin phenomenon [50]. Cao *et al*. characterize the antibody repertoires induced by Omicron BA.2 and BA.5 breakthrough infection. They find a reduction in the diversity of neutralizing antibody binding sites and an increase in the proportions of non-neutralizing antibody clones due to humoral immune imprinting [11]. The next-generation of SARS-CoV-2 vaccines should redirect immunity to epitopes associated with immune evasion [51].

The trivalent vaccine DBA2BA5 contains more epitopes than monovalent and bivalent RBD vaccines. After priming with the mRNA vaccines or other prototype strain-based COVID-19 vaccines, boosting with the DBA2BA5 vaccine may potentially elicits hybrid immune responses. Due to the effect of original antigenic sin, the Delta variant, which is antigenically similar to the prototype strain, can elicit memory responses induced by prior vaccination, with limited cross-reactivity to Omicron sub-variants. Meanwhile, the BA.2 and BA.5 RBDs can effectively stimulate naïve B cells and produce diverse antibody responses. Therefore, the epitopes on the DBA2BA5 vaccine would synergistically shape the induced immune responses. The effect of a booster dose of such an RBD heterotrimer vaccine and long-term durability studies will be further explored, which will guide the future design and use of SARS-CoV-2 vaccines.

## Materials and methods

### Ethics statement

Animal studies were approved by the Committee on the Ethics of Animal Experiments of the Institute of Microbiology, Chinese Academy of Sciences (IMCAS), and conducted in compliance with the recommendations in the Guide for the Care and Use of Laboratory Animals of the IMCAS Ethics Committee. The mice challenge experiments were conducted under animal biosafety level 3 (ABSL3) facility in Chinese Center for Disease Control and Prevention (China CDC), which were approved by the Ethics Committee of the National Institute for Viral Disease Control and Prevention, China CDC.

### Protein expression and purification

RBD homotrimer of SARS-CoV-2 prototype (PPP) was three RBD (S protein 319–537, GISAID: EPI_ISL_402119) connected as tandem repeat. The prototype-Delta-Omicron BA.1 heterotrimer (PDO) was one prototype RBD (S protein 319–534), one Delta RBD (S protein 320–534, GISAID: EPI_ISL_2020954) and one Omicron BA.1 RBD (S protein 317–534, GISAID: EPI_ISL_6640916) tandemly connected. The Delta-BA.2-BA.5 heterotrimer (DBA2BA5) was one Delta RBD (S protein 319–534, GISAID: EPI_ISL_2020954), one Omicron BA.2 RBD (S protein 317–531, GenBank: UVN39797.1) and Omicron BA.5 RBD (S protein 315–532, GenBank: OP603961.1) tandemly connected. For each construct, signal peptide sequence was added to the N-terminus and a hexa-His tag was added to the C-terminus. The coding sequences were codon-optimized, synthesized and cloned into pCAGGS vector.

The PPP, PDO and DBA2BA5 proteins were expressed in Expi293F cells (Thermo Fisher Scientific). Five days post transfection, the supernatants were collected. Soluble proteins were purified by Ni affinity chromatography with a HisTrap HP column (Cytiva). The proteins were further purified by gel filtration chromatography with Superdex 200 Increase 10/300 GL (Cytiva) in PBS buffer (10mM Na_2_HPO_4_, 2 mM KH_2_PO_4_ pH 7.4, 137 mM NaCl, 2.7 mM KCl).

The SARS-CoV-2 prototype monomeric RBD, Delta monomeric RBD, Omicron BA.1 monomeric RBD and prototype RBD-dimer proteins were prepared as previously described [18].

### Mouse

Specific pathogen-free (SPF) female BALB/c mice aged 6–8 weeks were purchased from Beijing Vital River Laboratory Animal Technology Co., Ltd. (licensed by Charles River). They were housed under SPF conditions in the laboratory animal facilities at Institute of Microbiology, Chinese Academy of Science (IMCAS). Mice were housed with 6 companions per cage. All animals were allowed free access to water and standard chow diet and provided with a 12-hour light and dark cycle (temperature: 20–25°C, humidity: 40%-70%).

### Analytical ultracentrifugation

Sedimentation velocity experiments were carried out using the ProteomeLab XL-I analytical ultracentrifuge (Beckman Coulter, Brea, CA). A volume of 380 μL of protein sample (0.6–0.8mg/mL) and 400 μL of matching buffer (10 mM Na_2_HPO_4_, 2 mM KH_2_PO_4_, pH 7.4, 137 mM NaCl, 2.7 mM KCl) were injected into appropriate channels of 12 mm double sector aluminum epoxy cells with sapphire windows. Solutions were centrifuged (39,000 rpm, 20°C) in an 60Ti rotor for 8 hours. Scans were collected at 280 nm, with 3 minutes elapsed between each scan. Data were analyzed using the continuous sedimentation coefficient distribution c(s) model in SEDFIT software.

### Surface plasmon resonance (SPR) assay

The SPR assays were carried out using a BIAcore 8k (Cytiva) at 25°C. The buffer for proteins used for kinetic analyses were PBST (10mM Na_2_HPO_4_, 2 mM KH_2_PO_4_ pH7.4, 137 mM NaCl, 2.7mM KCl, 0.005% Tween20). Prototype, Delta and Omicron BA.1 monomeric RBD proteins and PPP and PDO RBD-trimers were diluted with sodium acetate pH 5.0 and immobilized on a CM5 chip with the standard EDC/NHS coupling method. Gradient concentrations of hACE2 protein and antibody’s fab proteins were prepared and flowed over the chip surface with single cycle method. Data were collected over time and 10 mM Glycine pH 1.5 was used to regenerate the sensor. The binding kinetics (binding affinity, *K*_D_) were analyzed using 1:1 binding model with the software BIAevaluation Version 4.1 (GE Healthcare).

### Cryo-EM data collection and 3D reconstruction

The sample of Prototype RBD trimer bound to CB6 Fab (4.0 μL, 0.3 mg/mL) was applied to a Cu Quantifoil 1.2/1.3 holey carbon grid with glow discharged for 40 seconds and then blotted for 3 s with a humidity of 100% before being plunged into liquid ethane using a Vitrobot Mark IV (Thermo Fisher). Then the prepared grids were transferred to a 300 kV Titan Krios transmission electron microscope equipped with Gatan K3 detector and GIF Quantum energy filter. Movies were collected at 130,000x magnification with a calibrated pixel size of 0.67 Å over a defocus range of -1.0 μm to -2.0 μm in super resolution counting mode with a total dose of 60 e-/Å2 using EPU (ThermoFisher Scientific) automated acquisition software.

Similarly, for sample of Prototype-Delta-Omicron chimeric RBD-trimer bound to CB6 Fab, an aliquot of 4 μL solution (0.5 mg/mL) was applied to glow-discharged Quantifiol R 2/1 holey carbon grids and blotted for 3 s with a humidity of 100% before being plunged into liquid ethane using a Vitrobot Mark IV (Thermo Fisher). The frozen grids were loaded onto a Titan Krios cryo-transmission electron microscope (Thermo Fisher) that is equipped with a BioQuantum energy filter (Gatan), operated at 300 kV for data collection. Automatic data collection was performed using EPU software. Movies were recorded with a K3 direct electron in a super-resolution counting mode at pixel size of 0.428 Å. The exposure was performed with a dose rate of 20 e-/pixel/s and an accumulative dose of 50 e-/Å2 for each movie which was fractionated into 36 sub-frames. The final defocus range of the datasets was approximately-(1.2–2.0) μm.

The detailed data processing workflow is summarized in S2 Fig. The drift correction of all stacks were performed with MotionCor2 [52] to generate 2 x binned images. Initial contrast transfer function (CTF) values for each micrograph were calculated with CTFFIND4.1 [53]. Micrographs with an estimated resolution limit worse than 6.0 Å were discarded in the initial screening. The subsequent image processing and reconstruction were performed using Relion-3.1 [54] and cryoSPARC [55].

For the complex of Prototype RBD trimer bound to CB6 Fab, dataset was mainly processed using cryoSPARC. 285,000 initial particles from 7,172 micrographs were picked and extracted with the box size of 480 pixels. After three rounds of iterative 2D classification, a clean set of 93,228 particles were selected to generate the initial models and heterogeneous refinement and resulted to four distinct volumes. Then one volume containing ~33.3% of total particles were subjected to two rounds of homogeneous refinement, which yielded a final density map at 11.24 Å resolution estimated by the gold-standard Fourier shell correlation (FSC) cut-off value of 0.143. The specific images processing and reconstruction were shown in S2A Fig.

For the complex of Prototype-Delta-Omicron chimeric RBD-trimer bound to CB6 Fab, 223,689 particles were picked from 1854 micrographs. Then the picked particles were extracted and subjected to three rounds of reference-free 2D classification in Relion. A clean dataset with 105,829 particles from good 2D classes were selected and the initial model was generated by cryoSPARC ab initio. Then the model was used as reference in Relion 3D classification. After the third round of 3D classification without applying symmetry, the predominant class containing a subset of 19,523 good particles. These particles were subjected to 3D refinement, which yielded a reconstruction at ~15 Å resolution as determined by the Fourier shell correlation (FSC) 0.143 cut-off value. The specific images processing and reconstruction were shown in S2B Fig.

Due to the fierce flexibility between RBDs, we could only obtain low resolution maps as described above. However, we fitted the structures of RBDs (Prototype RBD:6LZG, Delta RBD:7V8B, Omicron RBD:7WBL) and CB6 fab (7C01) into those two complex density maps using CHIMERA [56], which showed a high degree of matching.

### Immunization

The adjuvant SWE (Sepivac) is a squalene-in-water emulsion adjuvant comprised of a metabolizable oil (squalene 3.9%, w/v), sorbitan trioleate (0.47%, w/v), and polyoxyethylene (80) sorbitan monooleate (0.47%, w/v) dispersed in 10 mM citrate buffer at pH 6.5 [26,57].

The antigen proteins were diluted with PBS buffer, mixed with an equal volume of SWE adjuvant and emulsified. BALB/c mice were vaccinated via the intramuscular injection with the vaccine candidates (2 μg). Blood samples were collected at several timepoints which were indicated in figure legends.

### ELISA

Antigen-binding properties of murine sera were determined by ELISA. Briefly, 96-well plates (3590, Corning, USA) were coated over-night with 3 μg/mL of antigen proteins in 0.05M carbonate-bicarbonate buffer (pH 9.6). The plates were blocked in 5% skim milk in PBS. Serum samples from mice were serially diluted and added to each well. The plates were incubated for 2 hours and then washed. Then, the plates were incubated with goat anti-mouse IgG-HRP antibody (Easybio, BE0102-100) for one hour and then washed. The plates subsequently developed with 3,3’,5,5’-tetramethylbenzidine (TMB) substrate. Reactions were stopped with 2 M hydrochloric acid, and the absorbance was measured at 450 nm using a microplate reader (Multiskan FC, Thermo Fisher). The endpoint titers were defined as the highest reciprocal dilution of serum to give an absorbance greater than 2.5-fold of the background values. Antibody titer below the limit of detection was determined as half the limit of detection. In Figs 1B, 3B, 4B and 6C, we measured the antigen-binding IgG titer for each group by coating the antigen protein used for immunization. In S5 Fig, we measured the RBD-binding IgG titer by coating the RBD monomer protein as indicated within the figure.

### Pseudotyped virus neutralization assay

The pseudotyped viruses displaying SARS-CoV-2 variants spike proteins express GFP in infected cells. They were prepared as previously described [6]. Mice sera were 2-fold serially diluted and incubated with pseudotyped virus at 37°C for 1 hour. Then the mixture was transferred to pre-plated Vero cell monolayers in 96-well plates. After incubation for more than 15 hours, the transducing unit numbers were calculated on a CQ1 confocal image cytometer (Yokogawa). Fifty percent pseudovirus neutralization titer (pVNT_50_) was determined by fitting nonlinear regression curves using GraphPad Prism and calculating the reciprocal of the serum dilution required for 50% neutralization of infection. pVNT_50_ below the limit of detection was determined as half the limit of detection.

### ELISpot assay

To detect antigen-specific cellular immune responses induced by vaccine candidates, ELISpot assays were performed using mouse IFNγ, IL-2 and IL-4 ELISpot kits (Mabtech, Sweden) according to the manufacturer’s protocols. A peptide pool consisting of 15-18-mers (overlapping by 11 amino acids) and spanning the RBDs of SARS-CoV-2 prototype, Delta and Omicron BA.1 were synthesized. Mouse splenocytes were stimulated with the peptide pool (10 μg/ml individual peptide). The numbers of the spots were determined using an automatic ELISpot reader and image analysis software (Mabtech).

### SARS-CoV-2 challenge experiments

To evaluate the protective efficacies of the vaccine candidates PDO, the SARS-CoV-2 challenge experiments were performed with four VOCs including Delta, Omicron BA.1, Omicron BA.2 and Omicron BA.4. A flowchart was shown in Fig 4A. BALB/c were immunized with two doses of 2 μg PDO (SWE adjuvant) or sham. Serum samples were collected before challenge.

For SARS-CoV-2 Delta VOC challenge experiments, mice were anesthetized with isoflurane and intranasally transduced with 8×10^9^ TCID_50_ of recombinant adenovirus expressing hACE2 (Ad5-hACE2) to rapid generation of a mouse model [58,59]. Five days later, the transduced mice were intranasally infected with 1.6×10^4^ TCID_50_ of SARS-CoV-2 Delta VOC (NPRC 2. 192100004). For SARS-CoV-2 Omicron BA.1, Omicron BA.2 and Omicron BA.4 VOCs challenge experiments, mice were challenged directly without transduction of Ad5-hACE2. The mice were intranasally infected with 9×10^3^ TCID_50_ Omicron BA.1 (NPRC 2.192100009), 7×10^3^ TCID_50_ Omicron BA.2 (NPRC 2.192100010) or 3×10^3^ TCID_50_ Omicron BA.4 (NPRC 2.192100012). These mice were euthanized and necropsied three days after challenge. The turbinate bones and lung tissues were harvested for virus titer determination. All animal experiments with SARS-CoV-2 challenge were conducted under ABSL3 facility in China CDC.

### Virus titer determination

Mice lung tissues and turbinate bones were weighted and homogenized. Virus titers were determined as previously described [18]. Briefly, SARS-CoV-2-specific quantitative reverse transcription-PCR (qRT-PCR) assays were performed using a FastKing One Step Probe RT-qPCR kit (Tiangen Biotech, China) on a CFX96 Touch real-time PCR detection system (Bio-Rad, USA) according to the manufacturer’s protocol. A set of primers and probe was used for detecting viral genome of Delta variant, with sequences as follows: RNA-F, GACCCCAAAATCAGCGAAAT; RNA-R, TCTGGTTACTGCCAGTTGAATCTG; RNA-probe, ACCCCGCATTACGTTTGGTGGACC. For detecting Omicron sub-variants (BA.1, BA.2 and BA.4), the sequences of primers used were same as the above description, and the probe sequence was ACTCCGCATTACGTTTGGTGGACC.

### Quantification and statistical analysis

*K*_D_ values for SPR assays were calculated by the software BIAevaluation Version 3.0 (GE Healthcare) using 1:1 binding model. Pseudovirus neutralization titers were determined by fitting nonlinear regression curves using GraphPad Prism and calculating the reciprocal of the serum dilution required for 50% neutralization of infection. The values shown are GMT ± 95% CI. The pVNT_50_ and virus titers were analyzed with a linear model using GraphPad Prism. Details can be found in figure legends.

P-values were analyzed with two-tailed Mann Whitney test or two-tailed unpaired t test (ns, p > 0.05; *p < 0.05; **p < 0.01; ***p <0.001; ****p < 0.0001). Details can be found in figure legends.

## Supporting information

S1 Fig Alignment of SARS-CoV-2 RBD sequences. SARS-CoV-2 prototype, Delta, Omicron BA.1, BA.2 and BA.4/5 RBD sequences were aligned by ESPript 3 (https://espript.ibcp.fr/ESPript/ESPript/). The paired cysteines are labeled by green numbers. The prototype RBD contains S protein residues 319–537.
(TIF)

S2 Fig SPR diagram of RBD-monomer and RBD-trimer proteins bound to hACE2 and mAbs. Monomeric SARS-CoV-2 RBD proteins from prototype, Delta and Omicron BA.1 strains, PPP and PDO RBD-trimers bound to hACE2 and mAbs. The antigen proteins were immobilized on the CM5 chip and were tested for binding with gradient concentrations of hACE2 or mAb Fabs as indicated using single-cycle mode by BIAcore 8000. The binding profiles are shown with time (s) on the x-axis and response units (RUs) on the y-axis.
(TIF)

S3 Fig Cryo-EM analysis of the CB6 Fab in complex with PPP and PDO RBD-trimers. Flow chart of cryo-EM data processing, Euler angle distribution of the final reconstruction and the FSC curve for the reconstruction for the CB6 Fab in complex with PPP (A) and PDO (B).
(TIF)

S4 Fig Stability analysis of DBA2BA5 protein. Analytical ultracentrifugation assays were performed with the DBA2BA5 protein stored at 4°C for 0, 7, 14, 21 and 28 days, respectively.
(TIF)

S5 Fig Endpoint titers of RBD-binding IgG in murine sera. The sera-binding antibody activities to prototype, Delta, Omicron BA.1, BA.2 or BA.5 RBD monomer protein were tested by ELISA.
(TIF)

S6 Fig Neutralization assays against SARS-CoV-2 pseudotyped viruses. Murine antisera were tested for neutralization of a panel of pseudotyped viruses displaying D614G, Delta, Omicron BA.1, BA.2, BA.2.75, XBB, BA.4/5, BF.7 or BQ.1.1 spike, respectively. Fitted nonlinear regression curves were generated using GraphPad Prism software.
(TIF)

S7 Fig pVNT_50_ analysis of murine sera. The fold change was calculated as the ratio of neutralization GMTs against the original virus (D614G) and the other variants. The minus symbol indicates that the GMT against the variant is lower than the original virus.
(TIF)

S8 Fig Durability of antibody responses induced by the DBA2BA5 vaccine. A group of 6- to 8-weeks-old female BALB/c mice (n = 6) was vaccinated with three doses of DBA2BA5 protein (2 μg) adjuvanted with SWE. PBS plus adjuvant was given as the sham control. Blood samples were collected at 21 and 120 days after the last dose immunization. The pVNT_50_ of the sera were measured by a panel of pseudotyped viruses. The values are the GMT ± 95% CI. The horizontal dashed line indicates the LOD. p values were analyzed with two-tailed Mann-Whitney tests (ns, p > 0.05; *p < 0.05).
(TIF)
