# Supplementary figures and images for: Broad protective RBD heterotrimer vaccines neutralize SARS-CoV-2 including Omicron sub-variants XBB/BQ.1.1/BF.7
Source: PLoS Pathog. 2023 Sep 18;19(9):e1011659. doi: 10.1371/journal.ppat.1011659 (PMC10538664; doi:10.1371/journal.ppat.1011659)

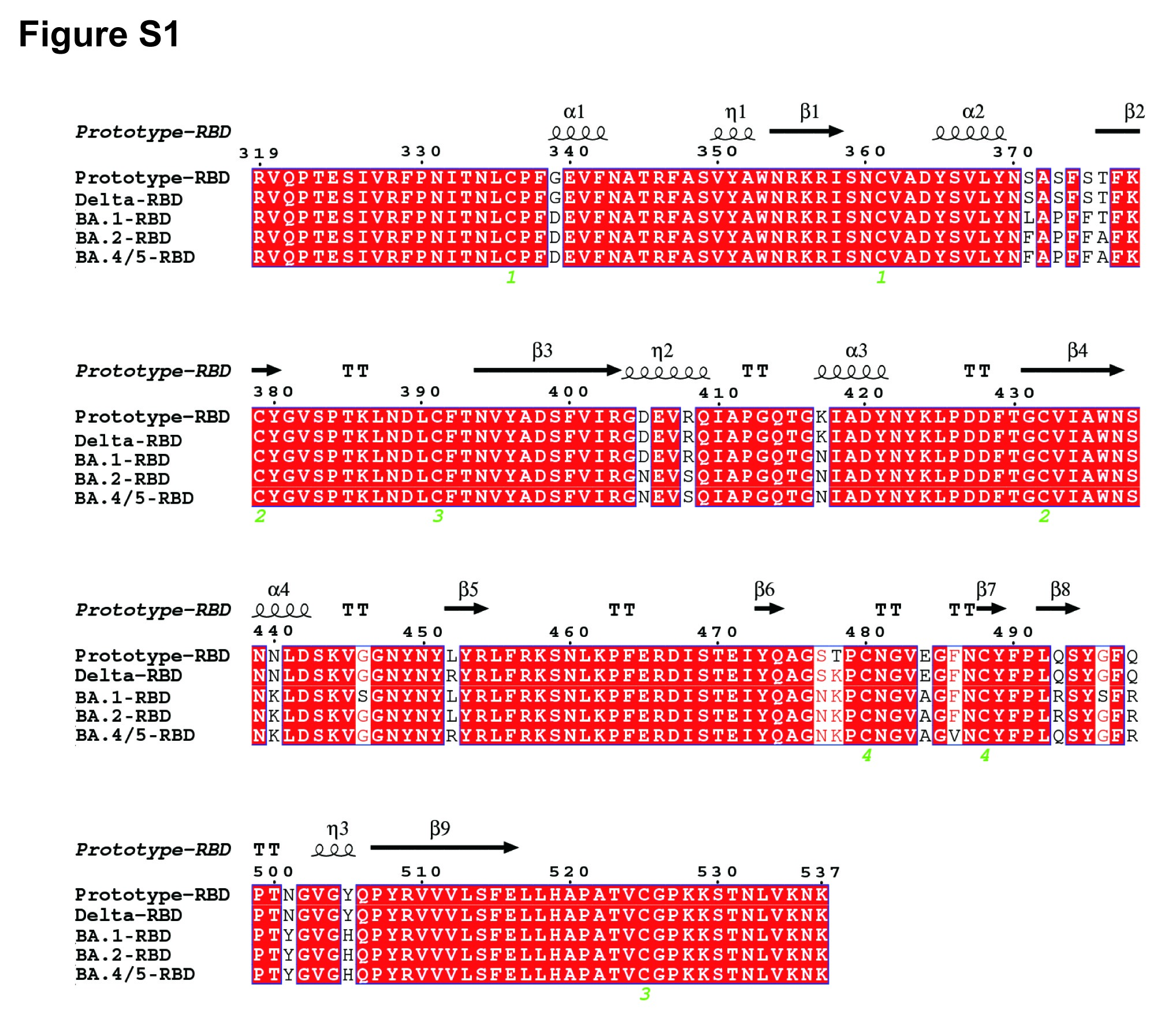

Supplement: S1 Fig — SARS-CoV-2 prototype, Delta, Omicron BA.1, BA.2 and BA.4/5 RBD sequences were aligned by ESPript 3 (https://espript.ibcp.fr/ESPript/ESPript/). The paired cysteines are labeled by green numbers. The prototype RBD contains S protein residues 319–537. (TIF) [file ppat.1011659.s001.tif]

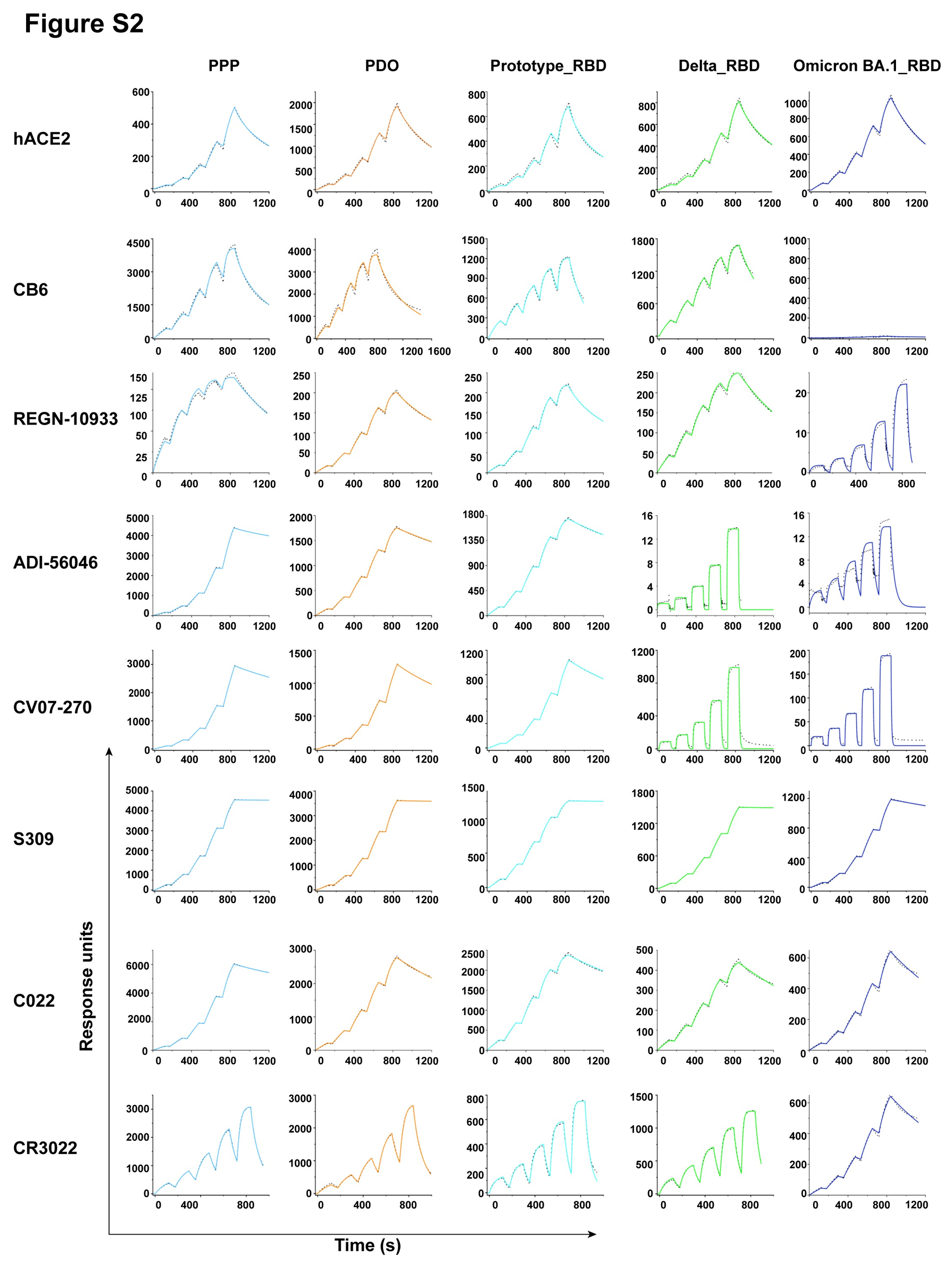

Supplement: S2 Fig — Monomeric SARS-CoV-2 RBD proteins from prototype, Delta and Omicron BA.1 strains, PPP and PDO RBD-trimers bound to hACE2 and mAbs. The antigen proteins were immobilized on the CM5 chip and were tested for binding with gradient concentrations of hACE2 or mAb Fabs as indicated using single-cycle mode by BIAcore 8000. The binding profiles are shown with time (s) on the x-axis and response units (RUs) on the y-axis. (TIF) [file ppat.1011659.s002.tif]

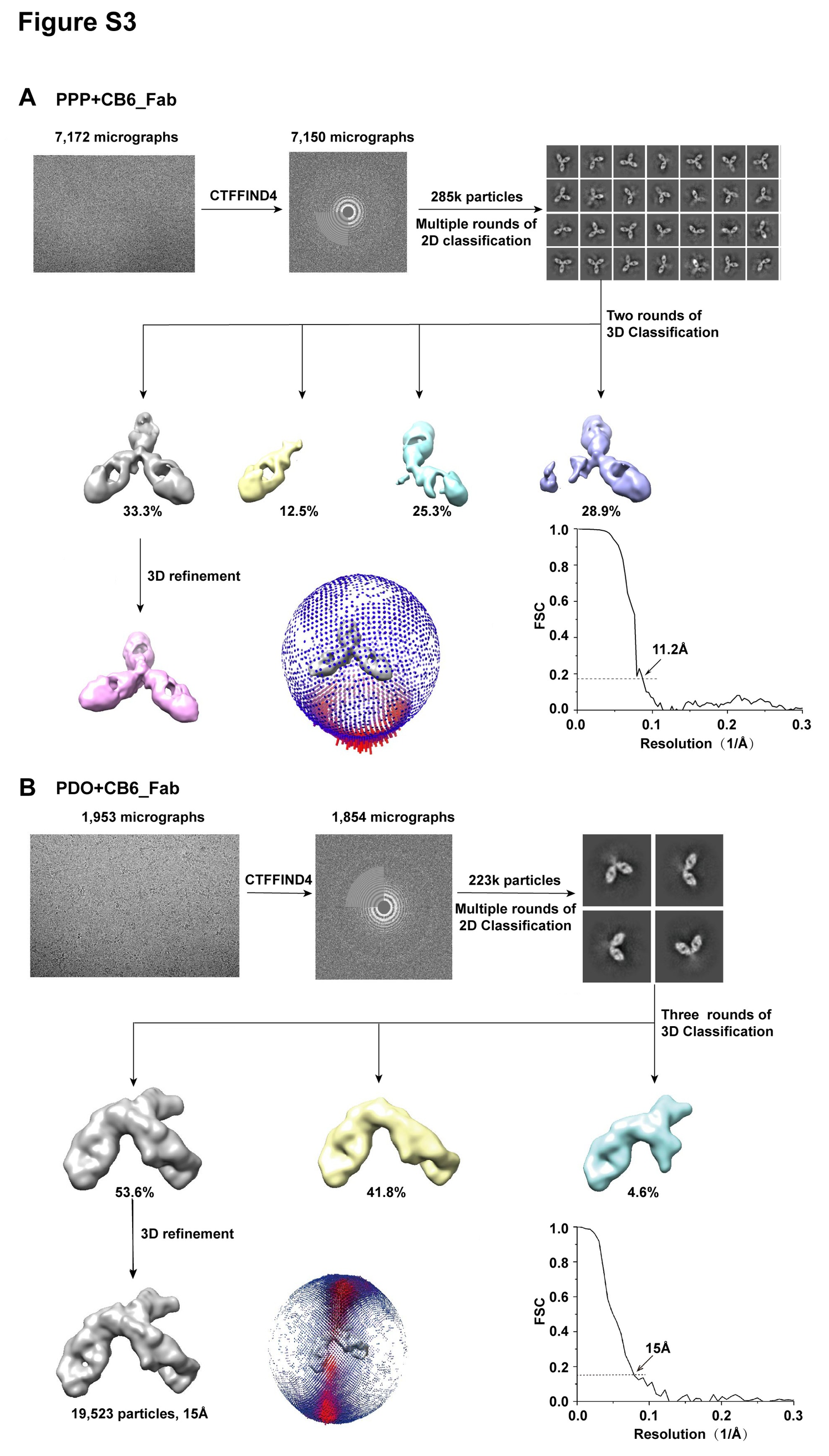

Supplement: S3 Fig — Flow chart of cryo-EM data processing, Euler angle distribution of the final reconstruction and the FSC curve for the reconstruction for the CB6 Fab in complex with PPP (A) and PDO (B). (TIF) [file ppat.1011659.s003.tif]

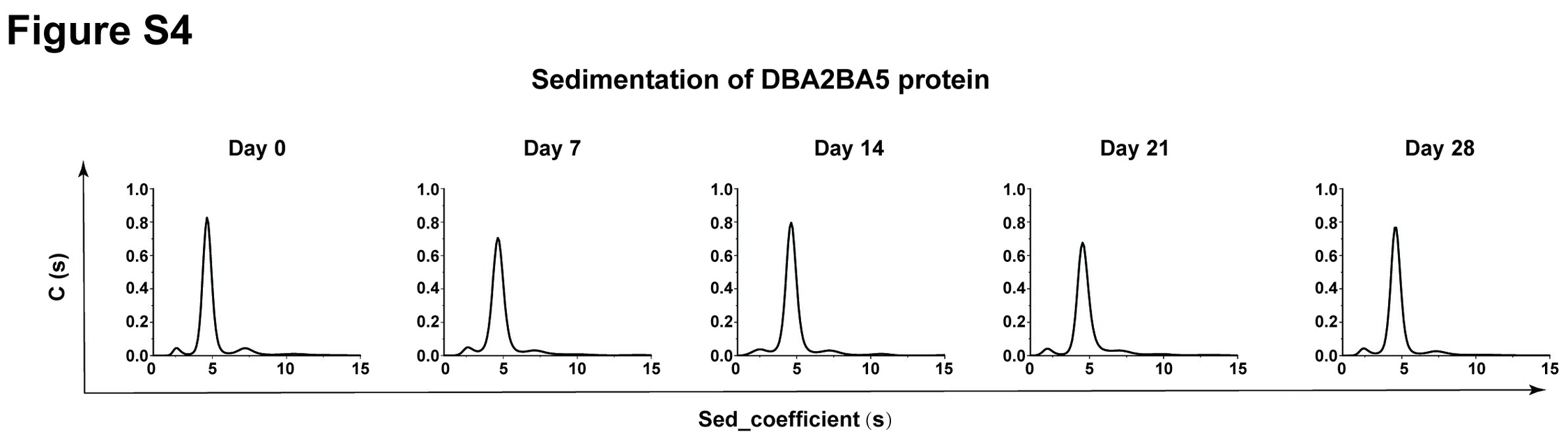

Supplement: S4 Fig — Analytical ultracentrifugation assays were performed with the DBA2BA5 protein stored at 4°C for 0, 7, 14, 21 and 28 days, respectively. (TIF) [file ppat.1011659.s004.tif]

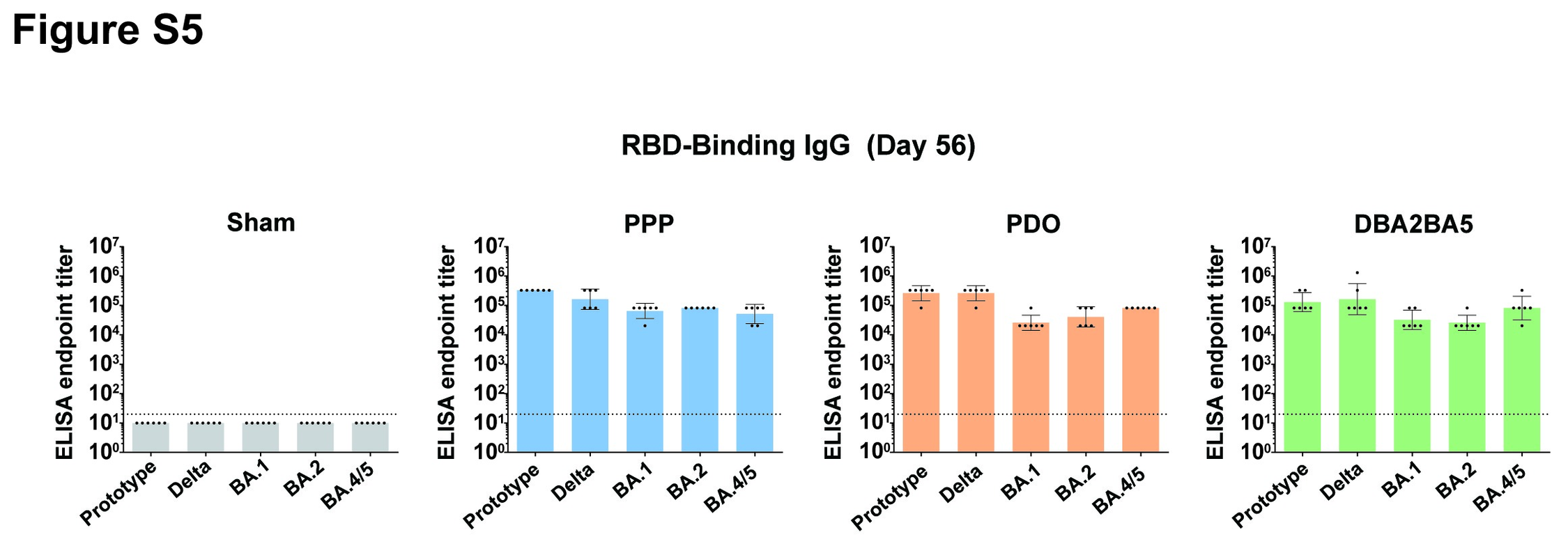

Supplement: S5 Fig — The sera-binding antibody activities to prototype, Delta, Omicron BA.1, BA.2 or BA.5 RBD monomer protein were tested by ELISA. (TIF) [file ppat.1011659.s005.tif]

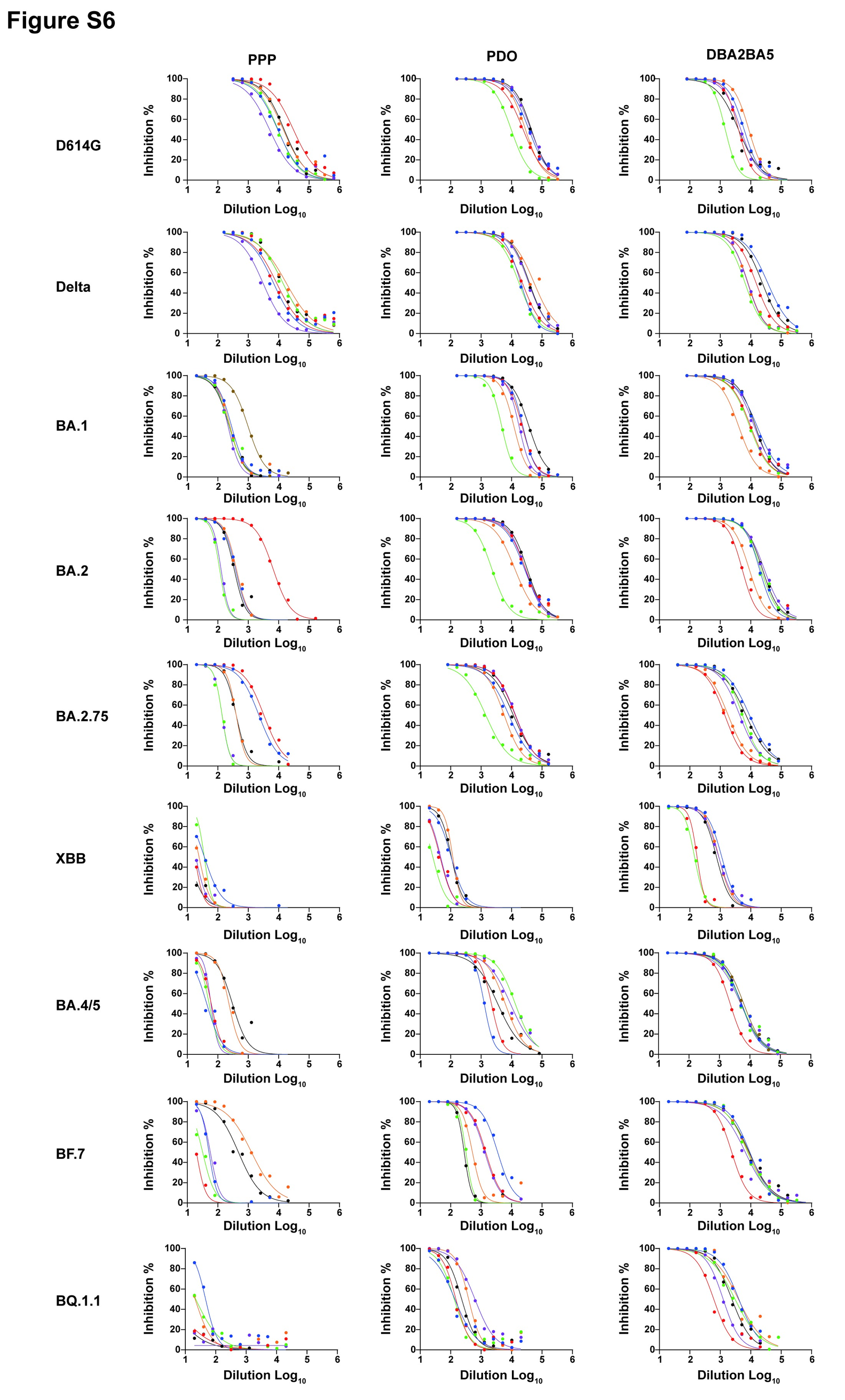

Supplement: S6 Fig — Murine antisera were tested for neutralization of a panel of pseudotyped viruses displaying D614G, Delta, Omicron BA.1, BA.2, BA.2.75, XBB, BA.4/5, BF.7 or BQ.1.1 spike, respectively. Fitted nonlinear regression curves were generated using GraphPad Prism software. (TIF) [file ppat.1011659.s006.tif]

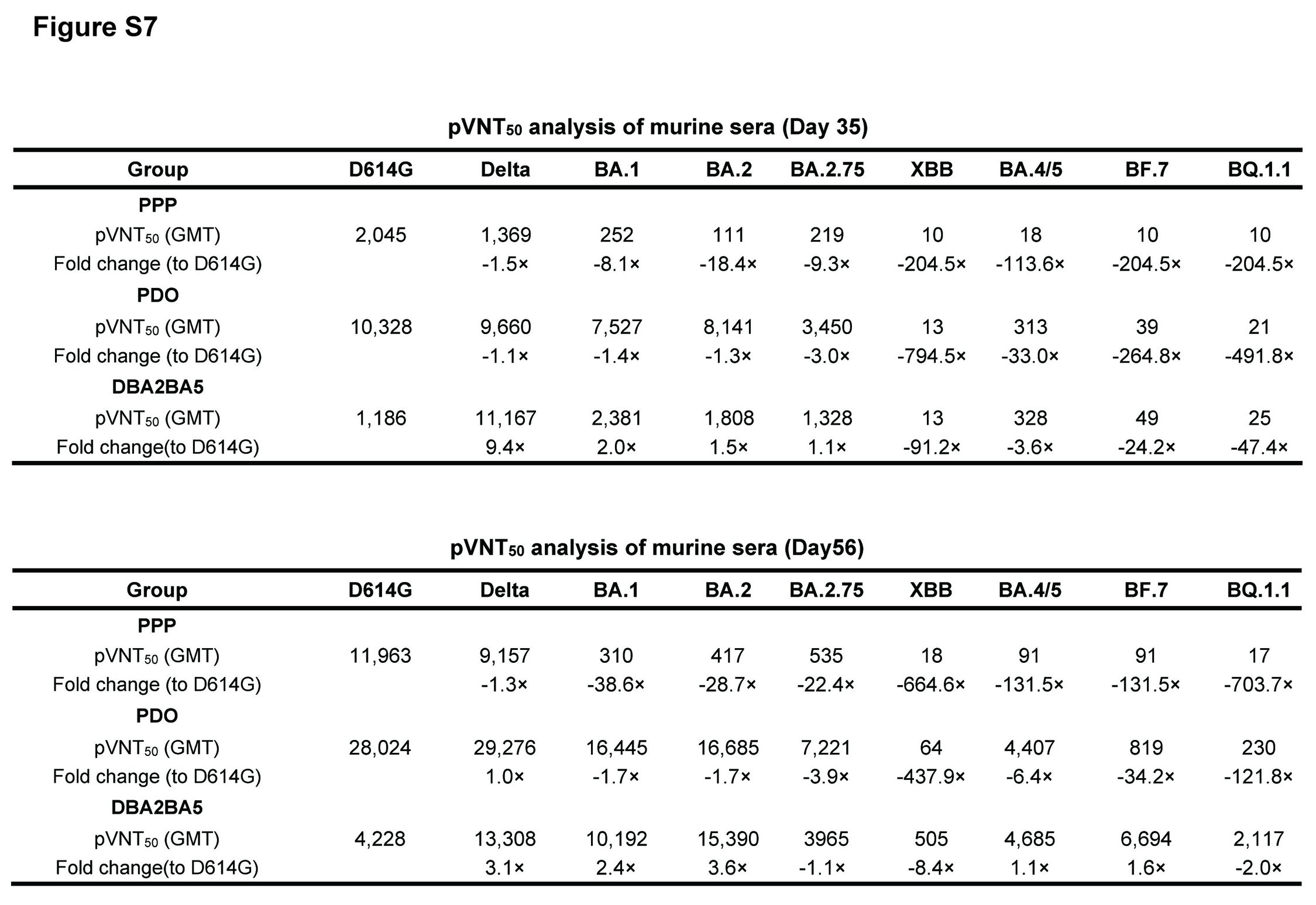

Supplement: S7 Fig — The fold change was calculated as the ratio of neutralization GMTs against the original virus (D614G) and the other variants. The minus symbol indicates that the GMT against the variant is lower than the original virus. (TIF) [file ppat.1011659.s007.tif]

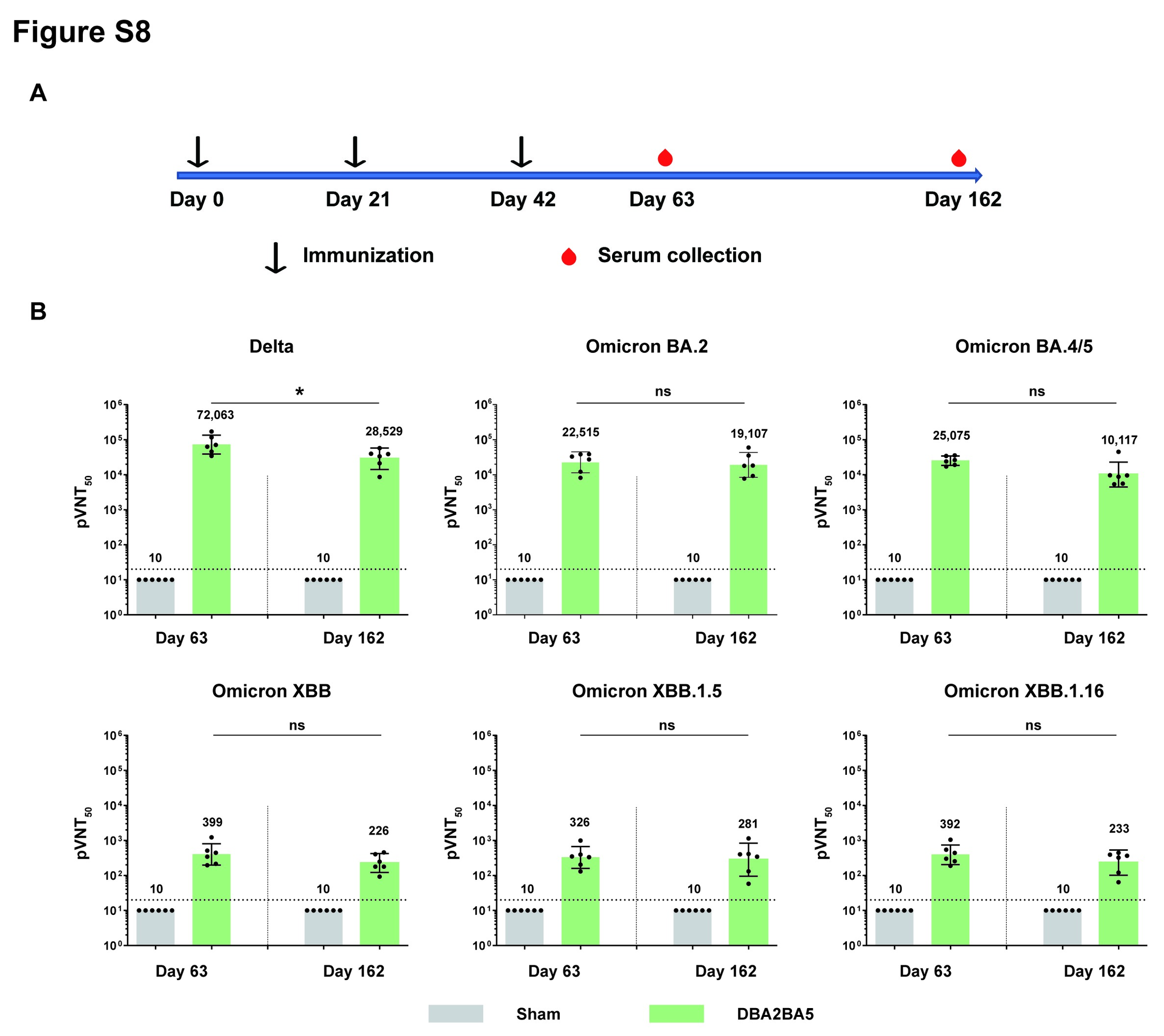

Supplement: S8 Fig — A group of 6- to 8-weeks-old female BALB/c mice (n = 6) was vaccinated with three doses of DBA2BA5 protein (2 μg) adjuvanted with SWE. PBS plus adjuvant was given as the sham control. Blood samples were collected at 21 and 120 days after the last dose immunization. The pVNT50 of the sera were measured by a panel of pseudotyped viruses. The values are the GMT ± 95% CI. The horizontal dashed line indicates the LOD. p values were analyzed with two-tailed Mann-Whitney tests (ns, p > 0.05; *p < 0.05). (TIF) [file ppat.1011659.s008.tif]
